# Genomic insights of aromatic hydrocarbon degrading *Klebsiella pneumoniae* AWD5 with plant growth promoting attributes: a paradigm of soil isolate with elements of biodegradation

**DOI:** 10.1007/s13205-018-1134-1

**Published:** 2018-02-07

**Authors:** Jina Rajkumari, L. Paikhomba Singha, Piyush Pandey

**Affiliations:** Soil and Environmental Microbiology Laboratory, Department of Microbiology, Assam University, Silchar, Assam 788011 India

**Keywords:** *Klebsiella pneumoniae*, Genome, Biodegradation, Hydrocarbons, Cleavage pathway, Heavy metal

## Abstract

This research employs draft genome sequence data of *Klebsiella pneumoniae* AWD5 to explore genes that contribute to the degradation of polyaromatic hydrocarbon (PAH) and stimulate plant growth, for rhizosphere-mediated bioremediation. Annotation analysis suggests that the strain AWD5 not only possess gene clusters for PAH utilization, but also for utilization of benzoate, fluorobenzoate, phenylacetate (*paa*), hydroxyphenylacetic acid (*hpa*), 3-hydroxyphenyl propionate (*mhp*). A comparative genome analysis revealed that the genome of AWD5 was highly similar with genomes of environmental as well as clinical *K. pneumoniae* isolates. The artemis output confirmed that there are 139 different genes present in AWD5 which were absent in genome of clinical strain *K. pneumoniae* ATCC BAA-2146, and 25 genes were identified to be present in AWD5 genome but absent in genome of environmental strain *K. pneumoniae* KP-1. Pathway analyzed using Kyoto Encyclopedia of Genes and Genomes enzyme database revealed the presence of gene clusters that code for enzymes to initiate the opening of aromatic rings. The polyaromatic hydrocarbon and benzoate degradation were found to be metabolized through ortho-cleavage pathway, mineralizing the compounds to TCA cycle intermediates. Genes for plant growth promoting attributes such as Indole acetic acid (IAA) synthesis, siderophore production, and phosphate solubilization were detected in the genome. These attributes were verified in vitro, including IAA (14.75 µg/ml), siderophore production (13.56%), phosphate solubilization (198.28 ng/ml), and ACC deaminase (0.118 mM α-ketobutyrate/mg) in the presence of pyrene, and also compared with results obtained in glucose amended medium. *K. pneumoniae* AWD5 enhanced the growth of *Jatropha curcas* in the presence of pyrene-contaminated soil. Moreover, AWD5 harbors heavy metal resistance genes indicating adaptation to contaminants. The study revealed the genomic attributes of *K. pneumoniae* AWD5 for its catabolic characteristics for different aromatic compounds, which makes it suitable for rhizoremediation of PAH-contaminated soil.

**Electronic supplementary material:**

The online version of this article (10.1007/s13205-018-1134-1) contains supplementary material, which is available to authorized users.

## Introduction

*Klebsiella pneumoniae* is a ubiquitous Gram-negative soil organism that lives in diverse environments. It is an important opportunistic pathogen; however, the organism is also known to be involved in nitrogen fixation (Lee et al. 2013). The catabolic capacity of *Klebsiella* strains to degrade hydrocarbons, including polyaromatic hydrocarbon (PAH), has been reported (Bhattacharya et al. 2003). There have been several reports on genome of *K. pneumoniae* justifying its virulence and most of the investigations have been focused on pathogenic strains. The genetic environment of antibiotic resistance elements such as OXA-232 and NDM-1 carbapenemases have been studied in detail in genomes of strain KP617 (Kwon et al. 2016) and NDM-1, beta-lactamases and 15 additional antibiotic resistance enzymes in strain ATCC BAA-2146 (Hudson et al. 2014). Similarly, virulence genes have been compared and described in detail among genomes of different pathogenic *K. pneumoniae* strains (Kwon et al. 2016).

*Klebsiella pneumoniae* is versatile for enduring in different microhabitats, which reflects in its genome. The genomic analysis of endophytic bacterium *K. pneumoniae* 342 revealed the presence of genes involved in colonization of growth in plants (Fouts et al. 2008). Liu et al. (2016) analyzed the genome of *Klebsiella* sp. D5A, which was isolated from the rhizosphere soil of tall fescue grown in oil-contaminated soil and showed that it promoted the growth of host plants in a petroleum-contaminated soil and enhanced phytoremediation efficiency. The hydrocarbon catabolic activities within the *Klebsiella* spp. have been reported, indicating that *Klebsiella* spp. are an important part of the oil-degrading microbial community (Rodrigues et al. 2009). Zhang et al. (2010) isolated *K. aquatica* sp. nov. from the activated sludge of a wastewater treatment plant in Zibo city, which was found to utilize naphthalene as the sole carbon and energy source for growth. In fact, *K. pneumoniae* SS12 and SS26 could grow in benzene, toluene, octane, and heptane (Survery et al. 2004), whereas *Klebsiella* sp. KCL-2 could mostly grow in n-alkanes but not in benzene, toluene, or xylene (Cha et al. 2000). Different species of *Klebsiella* appear to have substantial potential for the biodegradation of diverse pollutants, such as halogenated aromatic compounds (3, 5-dibromo-4-hydrobenzonitrile) (Mac Rae and Cameron 1985; McBride et al. 1986), nitroaromatic compounds (Kim and Song 2005), and 1, 2-dichloroethane (Mileva et al. 2008). *K. pneumoniae* strain PL1 could degrade 63.4% of pyrene and 55.8% of BaP in 10 days (Ping et al. 2014). *Klebsiella* sp. HL1 strain was reported for its capacity to degrade non- and mono-chlorinated dibenzofuran, and dibenzo-p-dioxin which encode dioxygenase genes (Fukuda et al. 2002).

Aromatic compounds are widely distributed into the environment and they are the main cause of water and soil pollution (Das and Chandran 2011). Petrochemical and petroleum products are one of the core energy sources and with increased human activities; there has been a sharp rise in environmental pollution levels (Njoku et al. 2009). Contaminations caused by petroleum products leave residual compounds which are harder to degrade. Decontamination of such places requires an advanced process termed as bioremediation; it takes the benefit of the catabolic microorganisms to detoxify pollutants (Kastner et al. 1998). Analysis of PAH catabolic genes in different species of bacteria gives useful information about the encoded enzymes, sequence–structure function relationships, evolution, and diversity of the catabolic genes (Chauhan et al. 2008). Aromatic hydrocarbon dioxygenases are the main enzymes considered to play key role in polyaromatic hydrocarbon that catalyze a double hydroxylation on two adjacent carbons of the substrate and ring-cleaving dioxygenases catalyze the opening of the ring of catecholic substrates (Kanaly and Harayama 2000). Oxygenases are necessary for the breakdown of PAH and dioxygenase genes were reported to be involved in degradation seem to be unique to a particular group of bacteria (Cerniglia 1992).

Various microorganisms of the taxonomic group such as *Sphingomonas* spp., *Pseudomonas* spp., *Burkholderia* spp., *Acinetobacter* spp., *Rhodococcus* spp., and *Mycobacterium* spp. (Uyttebroek et al. 2006) have been used for decontaminating aromatic compounds. Next-generation sequencing (NGS) has enabled to study whole-genome sequence of hydrocarbon degrading microorganisms. The study of these genomes provided global insights into the versatility of the bacterium, which enhanced biodegradation of pollutants. This provided the study of physiological and genetic background of the metabolic capability associated with pollutant degradation. Moreover, many complete and draft genome sequences relevant to biodegradation have been published which allowed the scientists to gain global insights into the evolutionary potential of specific microorganisms and their ability to bio-remediate polluted environments (Nierman and Nelson 2002; Buermans and Den Dunnen 2014). However, the genetic characteristics and the environment of hydrocarbon degrading genes in *K. pneumoniae* genomes have not been studied in detail.

Irrespective to the available information on *K. pneumoniae* as an agent for biodegradation of hydrocarbons, only a few genomes have been reported with industrial or environmental applications and the genomes of different *Klebsiella* strains have been studied and described with perspective of virulence. Here, the genomic features of *K. pneumoniae* AWD5 are described which was isolated from rhizospheric soil of a contaminated site from Assam, India (Rajkumari et al. 2017). *K. pneumoniae* AWD5 has excellent ability to degrade higher molecular weight (HMW) PAH; in addition, it also has plant growth promoting attributes like production of indole acetic acid (IAA), siderophore, and phosphate solubilization. *K. pneumoniae* AWD5 genome shows 4.8 Mb complex with 4155 protein-coding genes with function prediction and 375 without function prediction. In addition, the ability of the strain to degrade hydrocarbons is due to the presence of numerous dioxygenase genes which oxidatively catabolize aromatic rings. The genome also contains a complete carbohydrate metabolism pathway including glycolysis/gluconeogenesis, the tricarboxylic acid (TCA) cycle, and pyruvate metabolism. Therefore, the features of genome was analyzed and compared with other environmental and clinical isolates to realize the variations of a soil *K. pneumoniae* isolate. The mechanism of degradation of different hydrocarbons, pathways, and reactions was also determined.

## Materials and methods

### Growth condition

*Klebsiella pneumoniae* AWD5 was isolated from automobile waste-contaminated sites from Silchar, Assam. It was found to be facultative anaerobe, which could grow best around pH range of 4–8 in nutrient agar medium at 30 °C.

### Genome sequencing and annotation

The draft genome sequence of *K. pneumoniae* AWD5 was completed in September 2016 and submitted in GenBank for public access under the accession number MOXK00000000 in November 2016. The genomic DNA was purified from a pure culture of a single bacterial isolate of *K. pneumoniae* AWD5. DNA-purified libraries were quantified using qPCR according to the qPCR Quantification Protocol Guide (KAPA Library Quantification kits, Kapa Biosystems). Quality assurance of the genomic DNA preparation used for sequencing was assessed using the high-sensitivity DNA chip (Agilent Technologies, Waldbronn, Germany). The complete genomic DNA was sequenced using Next-Generation Sequencing System using Illumina HiSeq sequencing technology and assembled using CLC Genomics workbench v9.0. (Rajkumari et al. 2017). The annotation of protein-coding genes was provided by NCBI Prokaryotic Genome Annotation pipeline version 3.3 on NCBI website; further gene prediction and functional annotation were performed by Bacterial annotation system server 3 (BASys) and Integrated Microbial Genome-expert Review (IMG) pipeline. Genes responsible for degradation of aromatic compounds were annotated and the pathways of selective compounds were interpreted using KEGG pathway chart.

### Comparison of genomes

Graphical diagram for genome comparison of *K. pneumoniae* AWD5 was analyzed using Dot-plot in IMG-pipeline; it employs Mummer to generate dot-plot diagrams between two genomes. It uses input DNA sequences directly for comparing genomes with similar sequences (NUCmer). It uses six frame amino acid translation of the DNA input sequence (PROmer) for comparing genomes with dissimilar sequences (because DNA sequence is not highly conserved). Circular comparison of prokaryotic genomes is generated by BRIG (BLAST Ring Image Generator) version 0.95 following the manual, which shows similarity between a reference genome against other query sequence. Pairwise comparison of the genome sequence was done by Artemis Comparison Tool Release 13.0.0. The bacterial sequences were selected from IMG-pipeline for creating Artemis sequence file and it was further selected in ACT tool to draw the comparison view of the genome (Carver et al., 2012). Horizontal gene transfer was predicted using IslandViewer4 using the following methods—Integrated method, Island Path-DIMOB, and SIGI-HMM (Bertelli et al. 2017).

### Determination of PAH degradation using GC–MS

Bacterial inoculum was prepared by growing the bacterial isolates in peptone water. Cells were harvested and washed with phosphate buffer and resuspended in sterile water to give absorbance of 0.4 at 600 nm. The inoculum was then added to minimal medium Bushnell Hass (BH) broth containing 0.005% PAH (Pyrene, Chrysene, Benzo(a)pyrene) incubated at 30 °C and 140 rpm for 216 h. At the end of the experiment, the broth culture was taken with equal volume of ethyl acetate and the residual amount of PAH was extracted three times with acidification of the broth to pH 2–3 with concentrated HNO_3_ (Hesham et al. 2014).

### Enzyme activity assay

The enzyme activity was determined in the minimal medium (BH broth amended with 0.005% either pyrene or benzo(a)pyrene or chrysene as described (Singha and Pandey, 2017). From the broth culture, 10 ml cells (O.D._600_ = 0.5) were harvested by centrifugation (8000 rpm for 10 min.). The pellets were washed twice and resuspended in Tris buffer (50 mM). Cell-free extracts were prepared by treating the cells with GTE buffer, lysozyme, and sodium dodecyl sulfate (SDS) solution, incubated for 30 min at 37 °C with intermittent vortexing and centrifugation at 10,000 rpm for 10 min at 4 °C. The cell-free supernatant was used as crude enzyme for determining the enzyme activities in bacterial culture. Catechol 1, 2 dioxygenase (C-1,2D) and Catechol 2, 3 dioxygenase (C-2,3D) were assayed spectrophotometrically by measuring rate of production of metabolites from catechol as described (Silva et al. 2013).

### Plant growth promoting attributes and pot experiments

Freshly grown isolate was cultivated at 30 °C at 150 rpm for 7 days in either nutrient broth or minimal medium (BH broth amended with 0.005% pyrene as sole carbon source and energy) to determine IAA production (Gordon and Weber 1951). For the quantitative estimation of siderophore production, the cultures were inoculated in Guass medium, an iron-deficient medium containing (gl^−1^): K_2_HPO_4_, 6.0; KH_2_PO_4_, 3.0; MgSO_4_·7H_2_O, 0.2; (NH_4_)_2_SO_4_, 1.0; Succinic acid, 4.0 at 30 °C on a rotary shaker at 120 rev min^−1^ (Payne 1994), in two different carbon sources either glucose (2%) or pyrene (0.005%), respectively. Quantitative estimation of phosphate solubilization in the supernatant was estimated using the vanado-molybdate colorimetric method (Koenig and Johnson 1942) which was accomplished in Pikovskaya’s medium in two different culture conditions (either glucose or pyrene were used as carbon and energy source). 1-aminocyclopropane-1-carboxylic acid (ACC) deaminase activity was determined by measuring the production of α-ketobutyrate from the ACC cleavage by ACC deaminase (Penrose and Glick 2003). The freshly grown bacterial cultures were resuspended in 20 ml of Dworkin and Foster (DF) salts’ minimal medium supplemented with either (NH_4_)_2_SO_4_ or ACC (3.0 mM) as a sole nitrogen source. Another set of DF salts minimal medium, where pyrene (50 mg/L) instead of glucose as a sole carbon source and ACC as a nitrogen source were used, for determining bacterial ACC deaminase activity in response to pyrene were also determined. Pot trial experiments were performed to measure the effect of bacterial inoculation on growth of *J. curcas* pyrene-contaminated soil as described previously (Singha and Pandey 2017). The packed soil rite were spiked with various concentrations of pyrene (10, 20, 40, and 80 mg/kg), where trial without pyrene served as control. The seeds of *J. curcas* were surface sterilized by washing with distilled water and soaked in HgCl_2_ (0.1%) for 2–3 min and washed with distilled water. The seeds were bacterized as described (Singha and Pandey 2017). The seeds were sown directly to the pyrene-treated soils. All trials were experimented in triplicate.

### Statistical methods

The significance of experiments was tested using one-way ANOVA (*P* < 0.05). All data were processed using SPSS (version 22.0). Tukey’s test at 95% confidence interval was done using SPSS.

## Results

### General features of *K. pneumoniae* AWD5 genome

**Fig. 1:**
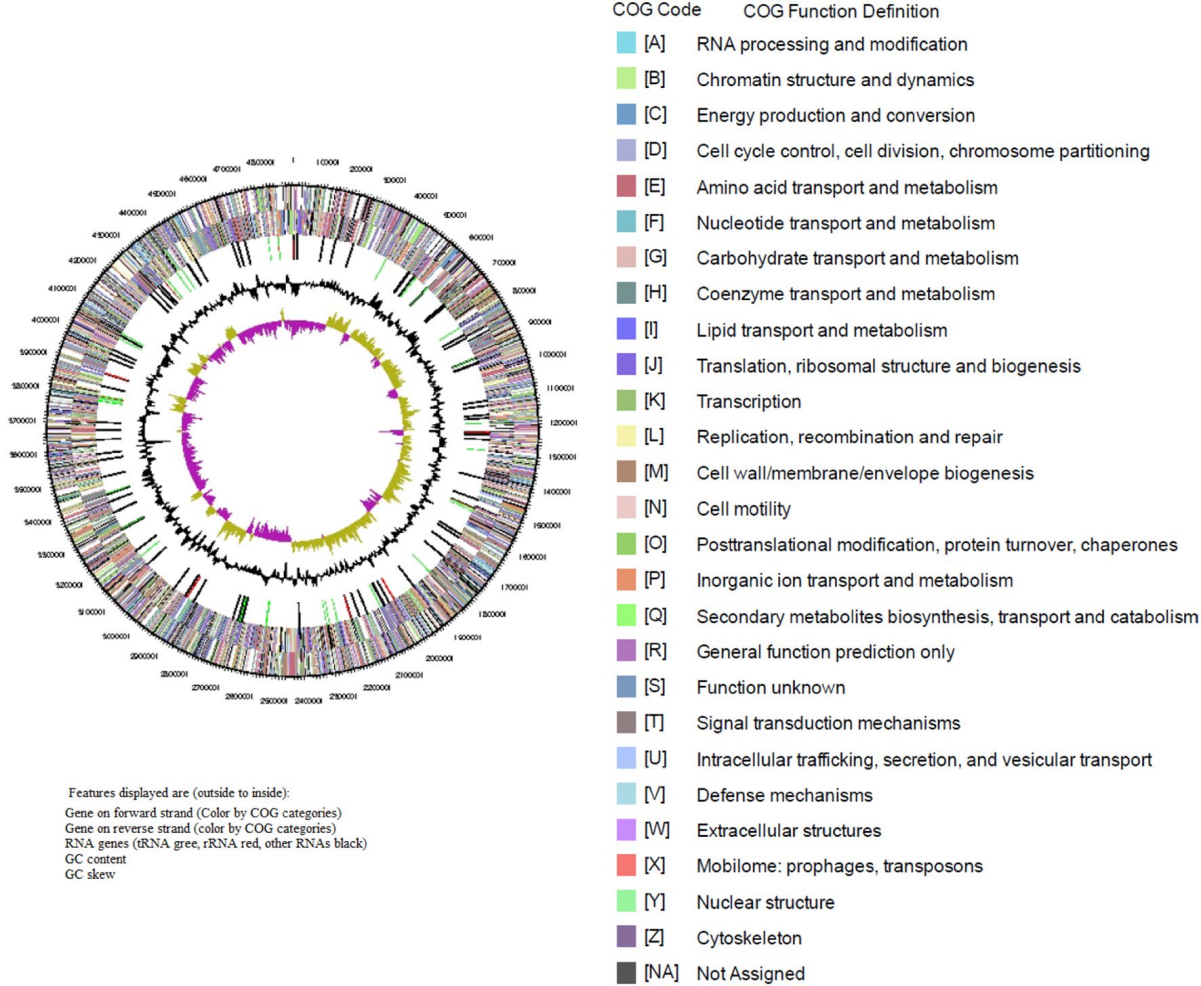
Circular chromosome map of *Klebsiella pneumoniae* AWD5

The draft genome of *K. pneumoniae* AWD5 consists of 4,807,409 bp of chromosomal DNA with G + C content of 58.18%. No plasmid was detected in AWD5. The general features of AWD5 genome are presented in Table 1 and Fig. 1, respectively.

**Table 1:** Genome statistics of *K. pneumoniae* AWD5

Features	Genome
DNA, total number of bases	4,807,409
DNA coding number of bases	4,364,072
DNA G + C content (%)	58.18
Tolal genes	4824
Protein-coding genes	4636
RNA genes	120
rRNA genes	25
5S rRNA	9
16S rRNA	8
23S rRNA	8
ncRNAs	14
tRNAs	81
Pseudo genes	68
Genes in internal clusters	1511
Genes with function prediction	4155
Genes with COGs	3842
Genes assigned to Pfam domains	4310
Genes coding signal peptides	438
Genes coding transmembrane proteins	1162

The protein-encoding genes (CDSs) were assigned to a putative function with the remaining annotated as hypothetical proteins. Among the 4530 CDSs, 3842 (81.35%) were assigned to 27 different clusters of orthologous groups (COGs) (Table 2).

**Table 2:** Number of genes in *K. pneumoniae* AWD5 associated with 27 general COG functional categories

Code	Gene count	% of total	Description
E	457	10.59	Amino acid transport and metabolism
G	513	11.89	Carbohydrate transport and metabolism
D	42	0.97	Cell cycle control, cell division, chromosome partitioning
N	47	1.09	Cell motility
M	215	4.98	Cell wall/membrane/envelope biogenesis
B	1	0.02	Chromatin structure and dynamics
H	230	5.33	Co-enzyme transport and metabolism
Z	1	0.02	Cytoskeleton
V	88	2.04	Defense mechanism
C	281	6.51	Energy production and conversion
W	26	0.6	Extracellular structures
S	216	5.01	Function unknown
R	343	7.95	General function prediction only
P	309	7.16	Inorganic ion transport and metabolism
U	60	1.39	Intracellular trafficking, secretion and vesicular transport
I	138	3.2	Lipid transport and metabolism
X	3	0.07	Mobilome: prophages, transposons
F	108	2.5	Nucleotide transport and metabolism
O	170	3.94	Posttranslational modification, protein turnover, chaperons
A	1	0.02	RNA processing and modification
L	121	2.8	Replication, recombination and repair
Q	115	2.67	Secondary metabolites biosynthesis, transport and catabolism
T	182	4.22	Signal transduction mechanisms
K	397	9.2	Transcription
J	250	5.8	Translation, ribosomal structure and biogenesis
–	881	18.65	Not in COG

The total is based on the total number of protein-coding genes in the annotated genome

### Comparative genome analysis of *K. pneumoniae* AWD5 genome

**Fig. 2:**
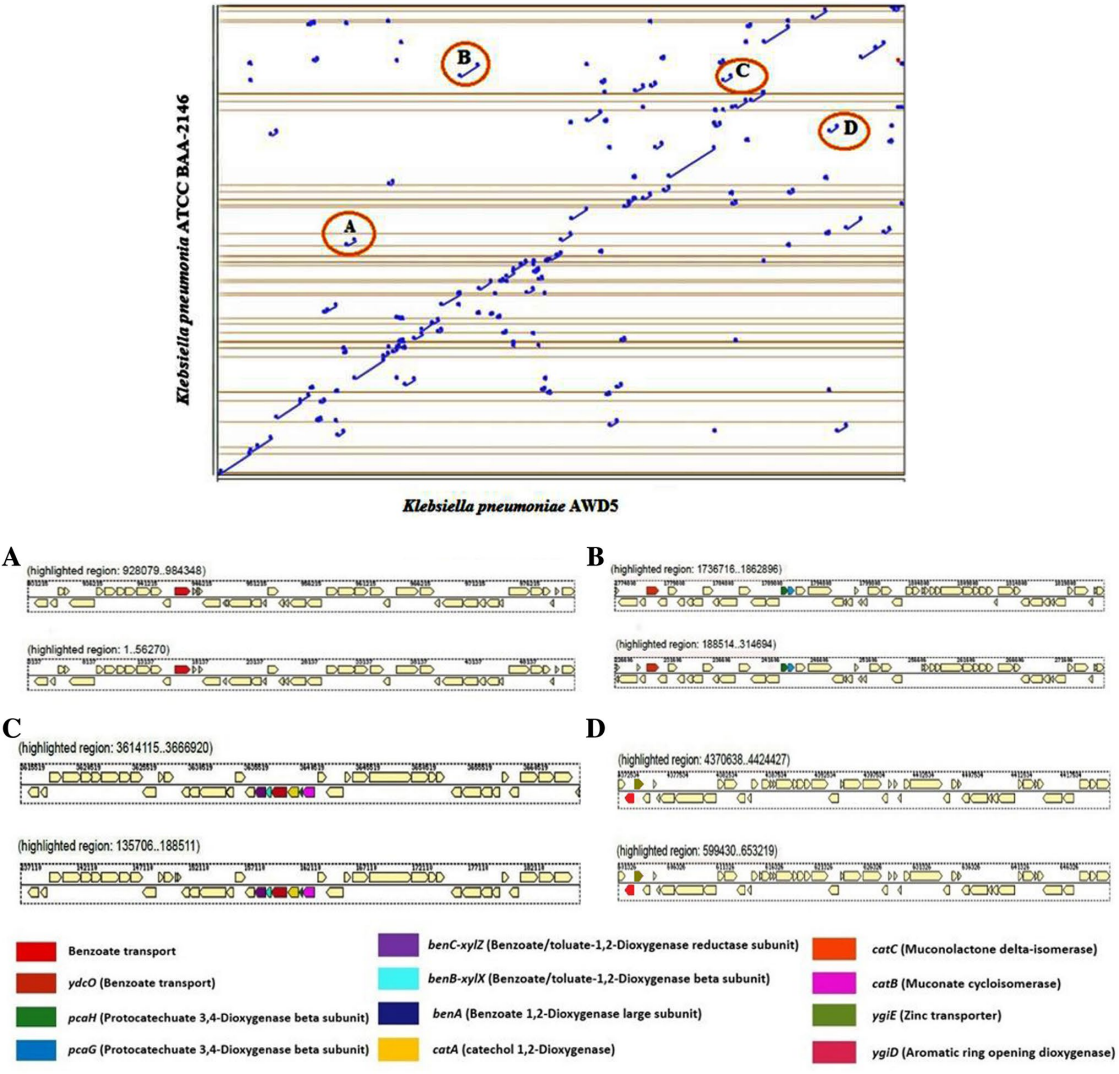
Dot plot of nucleotide sequence between *K. pneumoniae* AWD5 and *K. pneumoniae* ATCC BAA. Blue points signifies regions of similarity found on parallel strands (fplot); red points indicates similarity on antiparallel strand (rplot)

Genomes of *K. pneumoniae* AWD5 (X-axis) and *K. pneumoniae* ATCC BAA-2146 (Y-axis) were compared and graphically represented as a dot-plot (Fig. 2). It was interesting to note that several gene elements deflected from collinear arrangement. Few of such hydrocarbon degrading genes have been marked and elaborated in Fig. 2a, b, c, d, which indicate that they have high similarity percentage (Table S1, supplementary data). Careful comparisons of these four regions suggested the presence of set of similar genes arranged as non-syntenic blocks.

**Fig. 3:**
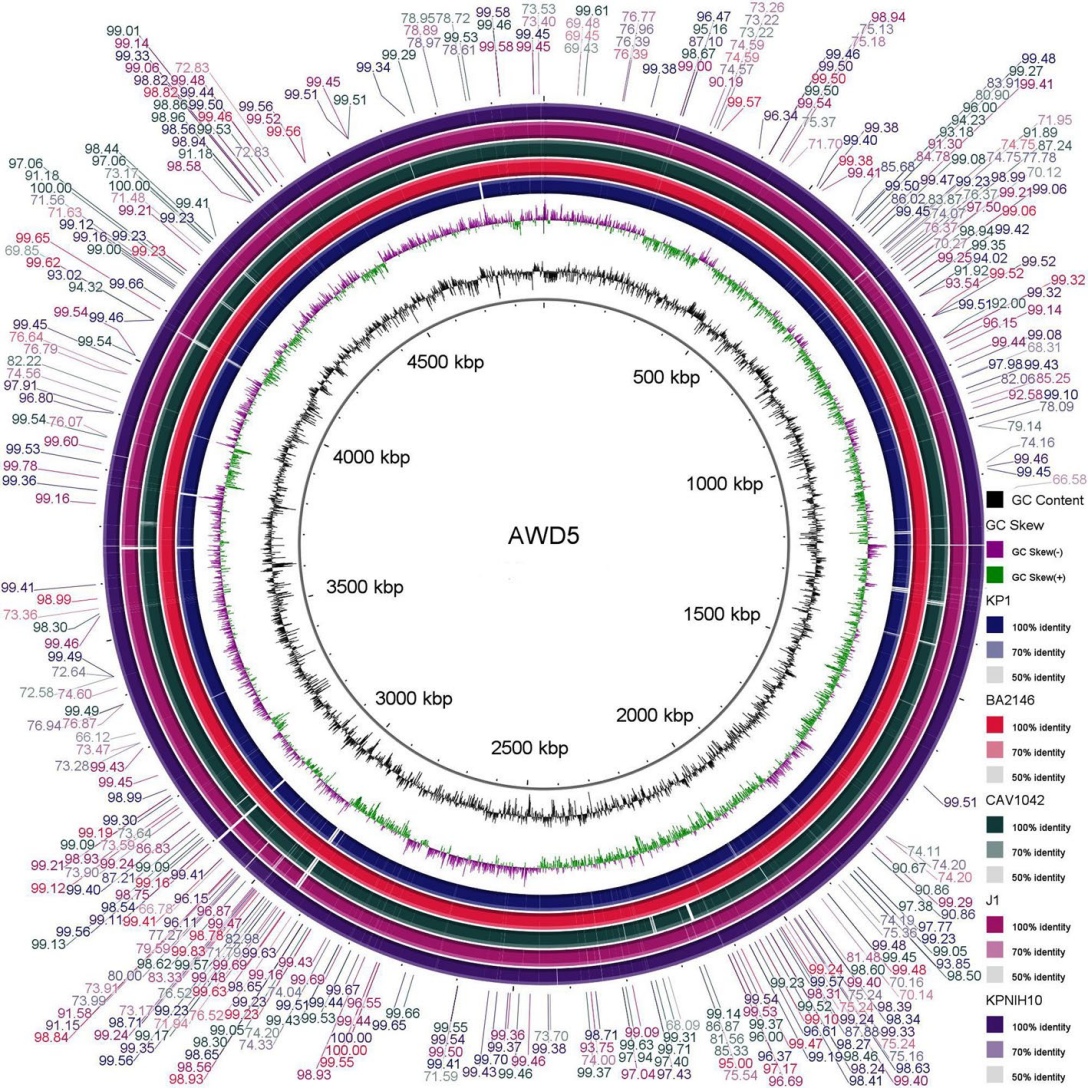
BRIG output image of a draft genome *K. pneumoniae* AWD5 against 5 other *Klebsiella* genome. The innermost rings show GC skew (green/purple) and GC content (black). The third innermost ring (blue colored) shows genome of *K. pneumoniae* KP-1, fourth ring gives (pink) genome of *K. pneumoniae* ATCC BAA-2146, fifth ring gives (green) genome of *K. pneumoniae* CAV1042, sixth ring gives (purple) genome of *K. pneumoniae* J1, and seventh ring gives (violet) genome of *K. pneumoniae* KPNIH10

The image generated by BRIG analysis provided the comparative and visual assessment of *K. pneumoniae* AWD5 genome against genome of five other *K. pneumoniae* strains. As represented in Fig. 3, the genome of AWD5 was at the center, where the innermost ring depicts GC content (black), and GC skew (green/purple). In fact, the query sequences were selected on the basis of similarity shown by BLAST search; accordingly, genome sequences of environmental strains (J1, KP1) as well as isolates of clinical relevance (BA2146, CAV1042, and KPNIH10) were selected to generate comparative image in BRIG. Five *K. pneumoniae* strains were selected from IMG database (Table 3). The shaded regions in BRIG output gave the similarity of the reference against query sequence (here AWD5). Interestingly, AWD5 genome was almost 100% similar with BA2146 (*K. pneumoniae* ATCC BAA-2146) and was found to have minor variations with KPNIH10 (*K. pneumoniae* KPNIH10) (Fig. 3). The findings were further substantiated by comparing the genome statistics of *K. pneumoniae* strains used for BRIG analysis, retrieved from http://img.jgi.doe.gov (Table 3).

**Table 3:** Summary of genome statistics of *K. pneumoniae* used for comparative analysis

Genome features^a^	*K. pneumoniae* AWD5	*K. pneumoniae* ATCC BAA 2146	*K. pneumoniae* J1	*K. pneumoniae* KP1	*K. pneumoniae pneumoniae* KPNIH10	*K. pneumoniae pneumoniae* CAV1042
Taxon ID	2711768630	2529293104	2687453357	2602041576	2519103053	2721755828
DNA total number of bases	4,807,409	5,680,367	5,406,866	5,131,085	5,716,118	5,752,260
DNA coding number of bases	4,364,072	4,992,944	4,771,385	4,555,270	4,943,008	5,092,850
G + C content	58.18%	57.02%	57.24%	57.60%	57.22%	56.83
Total genes	4824	5636	5251	4919	5730	5699
Protein-coding genes	4636	5552	5039	4755	5620	5486
RNA genes	120	84	212	164	110	213
tRNA genes	81	77	88	74	84	86
Other RNA genes	86	–	99	81	–	102

^a^*K. pneumoniae* strains used in BRIG analysis were retrieved from IMG database (http://img.jgi.doe.gov)

**Fig. 4:**
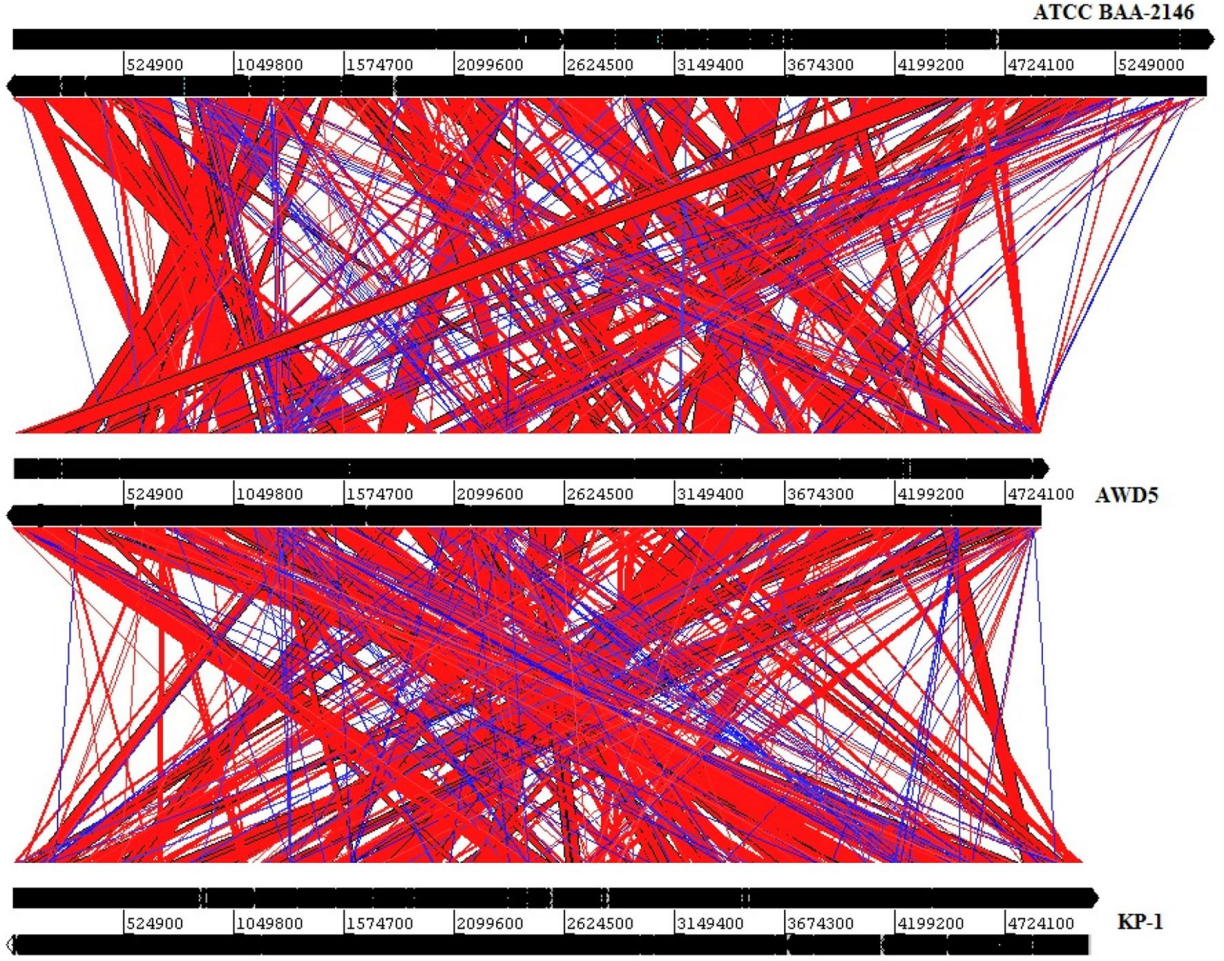
ACT illustration for three-way pairwise genome comparison of *K. pneumoniae* AWD5 genome with *K. pneumoniae* ATCC BAA-2146 (upper panel), and *K. pneumoniae* KP-1 (lower panel). The regions of homologous sequences are linked by block; red gives the level of similarity in the same orientation; while blue color links the similar regions in reverse orientation

The Artemis Comparison Tool (ACT) illustration generated a three-way comparison of *K. pneumoniae* AWD5 (in middle row) with a reference genome *K. pneumoniae* ATCC BAA-2146 (on top) and a similar genome *K. pneumoniae* KP-1 (below), isolated from environment (Fig. 4). Expanding the Artemis output for genome comparison, it was suggested that there were 139 different genes present in AWD5 which were absent in ATCC BAA-2146, which also included heavy metal (cobalt resistance operon *cbi*-gene cluster, copper stress-related genes—*copA cueO*, and copper responsive transcriptional regulator *cueR*, *copA*, etc.). This may be assumed to provide better competiveness to AWD5 in Cu- or Co-contaminated soil or other environmental habitats, as compared to clinical isolate ATCC BAA-2146. AWD5 also have *pdu* gene cluster for propanediol metabolism, which was absent in ATCC BAA-2146. Furthermore, 25 genes were identified to be present in AWD5 genome but absent in KP-1 genome, majority of which comprised carbohydrate metabolism.

A comparative summary of selected annotated gene functions of *K. pneumoniae* AWD5 genome with that of *K. pneumoniae* ATCC BAA-2146 (clinical, reference strain) and *K. pneumoniae* KP-1 (environmental isolate) genome is given in (Table S1). All three genomes had several genes for phenyl acetic acid metabolism, hydroxy phenyl propionate degradation, 3, 4-dihydroxyphenylacetate degradation, and aromatic hydrocarbon degradation, including *catA*, *catB*, *catC*, and *benB, benC*, drug metabolism-cytochrome p450, transport and catabolism genes, and multi-drug resistance protein. ATCC BAA-2146 did not have *mhp* operon, while KP-1 did not have *hpc* operon. Isolated AWD5 has both the operons. Furthermore, *K. pneumoniae* AWD5 was found to have drug-resistant genes and 12 bacterial infectious disease-related gene, while KP-1 and ATCC BAA-2146 were found to have 41 and 39 bacterial infectious disease-related genes, respectively.

### Degradation of PAH by AWD5 and its genetic elements

**Fig. 5:**
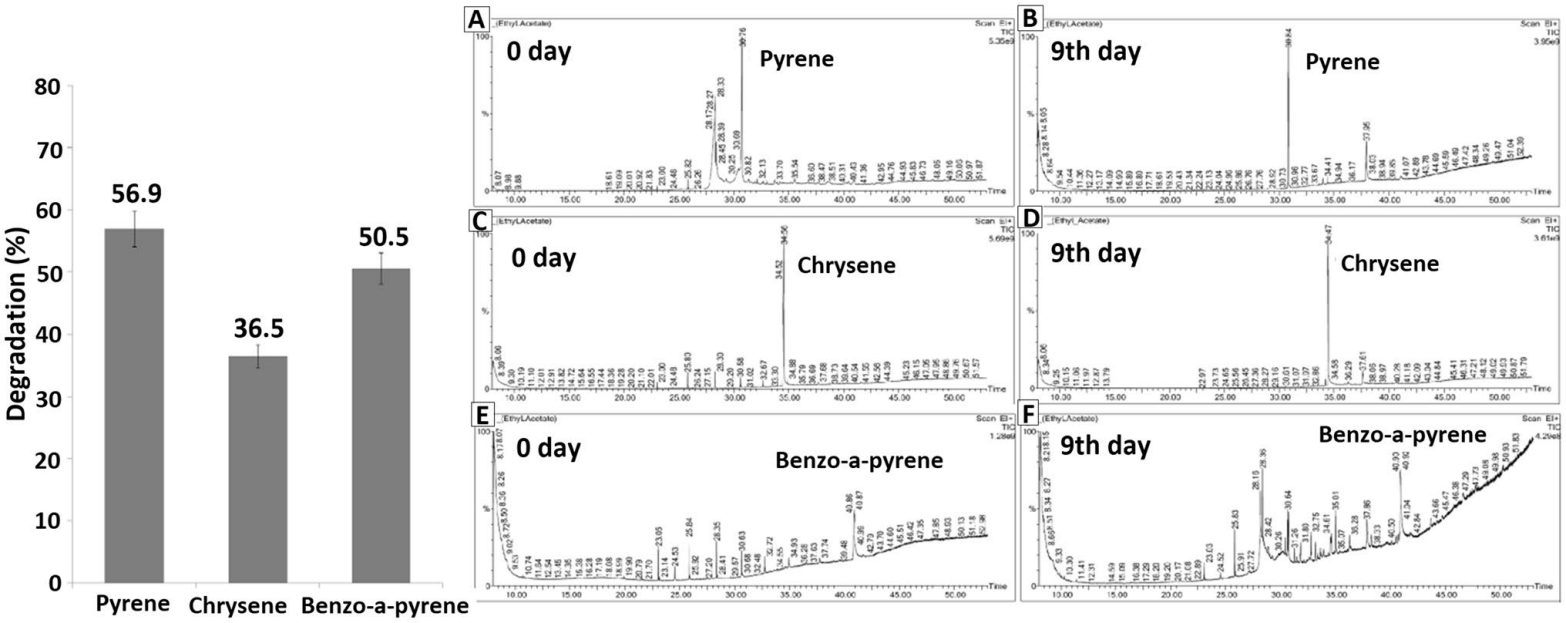
Degradation (%) of pyrene, chrysene, and benzo(a)pyrene after 9 days of incubation by AWD5

**Fig. 6:**
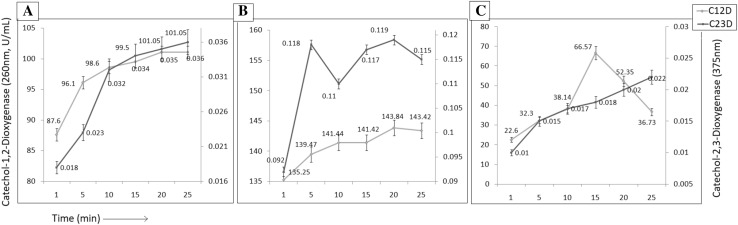
Enzyme activity of C-1,2D and C-2,3D in BH medium amended with Pyrene (**a**), Benzo(a)pyrene (**b**), and Chrysene (**c**)

**Fig. 7:**
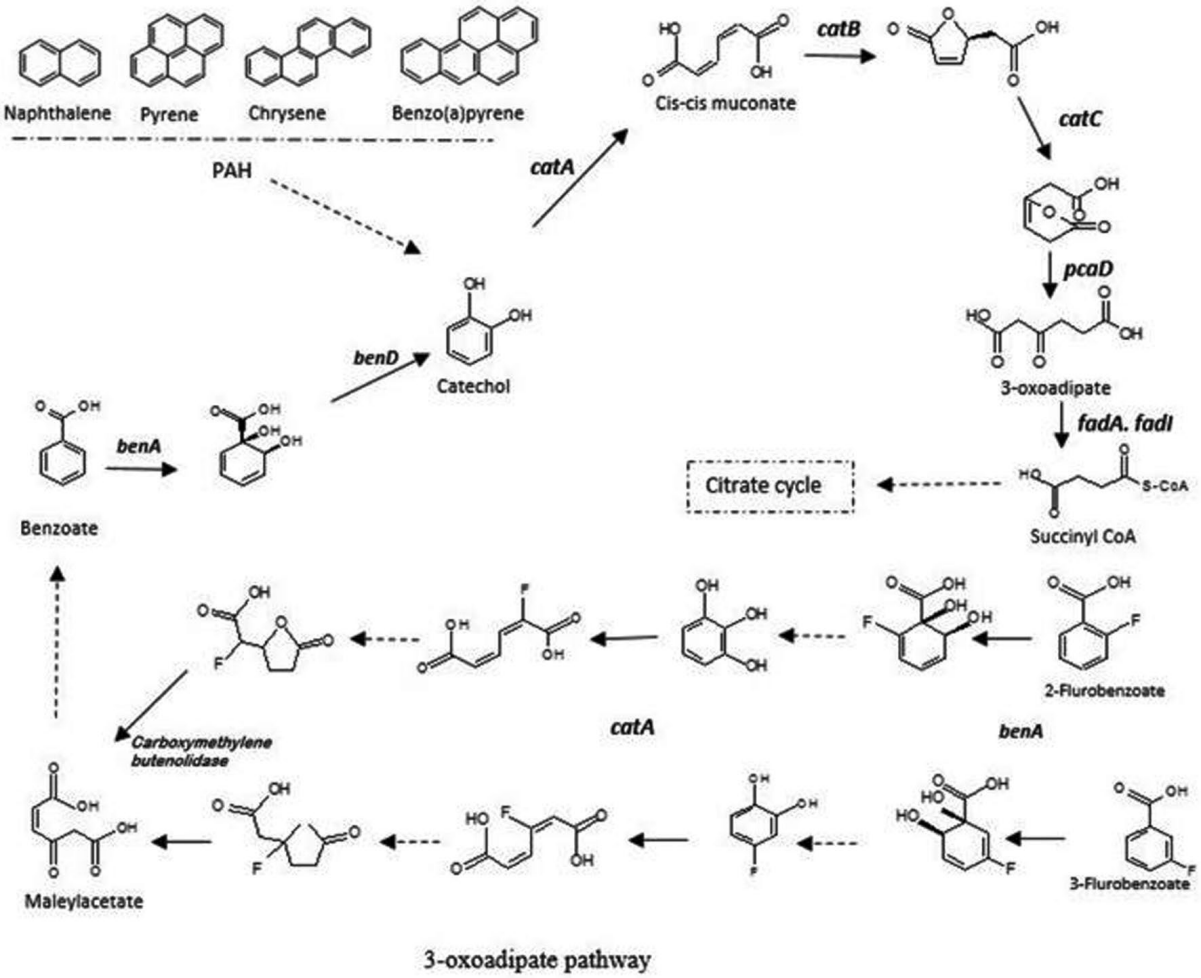
Catechol ortho-cleavage 3-oxoadipate pathway for PAH, Benzoate, 2- and 3-Fluorobenzoate degradation with intermediates and respective genes present in genome of *K. pneumoniae* AWD5

*Klebsiella pneumoniae* AWD5 was found to degrade pyrene (56.9%), chrysene (36.5%) and benzo(a)pyrene (50.5%), respectively, after 9 days of incubation (Fig. 5) as confirmed by in vitro experiments. C-1,2D and C-2,3D activities were recorded at different time intervals, in the presence of different PAH, and were found better, when benzo(a)pyrene was used as substrate, followed by pyrene and chrysene (Fig. 6). The highest activity of C-1,2D was observed in benzo(a)pyrene (143.84 U/mL) followed by in pyrene (101.05 U/mL) and lowest (66.57 U/mL) in chrysene amended medium. Phthalate and catechol were detected after 9 days which confirms the proposed pathway (Fig. 7).

The genome of AWD5 contains several genes for degradation of aromatic compounds. The genome of *K. pneumoniae* AWD5 is found to carry aromatic ring-opening dioxygenase (*ygiD*), extradiol catechol dioxygenase, catechol 1,2-dioxygenase (*catA*), muconolactone (*catB*), muconolactone δ-isomerase (*catC*), catechol 2,3-dioxygenase, toluate 1,2-dioxygenase, and 3,4-dihydroxyphenylacetate 2,3-dioxygenase (Fig. 11a). In addition, benzoate dioxygenase large subunit (*benA*) and small subunit (*benB*) were also present which is responsible for initiation of benzoate degradation. The key ring cleavage dioxygenase enzyme of protocatechuate (3, 4-dihydroxybenzene, PCA) degradation is found in the genome. Protocatechuate was cleaved between their two hydroxyl groups by protocatechuate 3, 4- dioxygenase in β-ketoadipate pathway (Harwood and Parales 1996). A benzoate transporter gene (*ydcO*), protocatechuate 3, 4-dioxygenase α-subunit (*pcaG*), protocatechuate 3, and 4-dioxygenase β-subunit (*pcaH*) were organized as an operon with transcription regulator and quinate dehydrogenase (*qumA*) (Fig. 11b). The strain AWD5 cleaved aromatic rings via intradiol ring cleavage by dioxygenases resulting in the formation of central intermediate catechol, leading to the formation of 3-oxoadipate and finally converted to TCA cycle intermediates (Fig. 7) (Cerniglia 1992; Eaton and Chapman 1992; Gibson and Parales 2000).

**Fig. 8:**
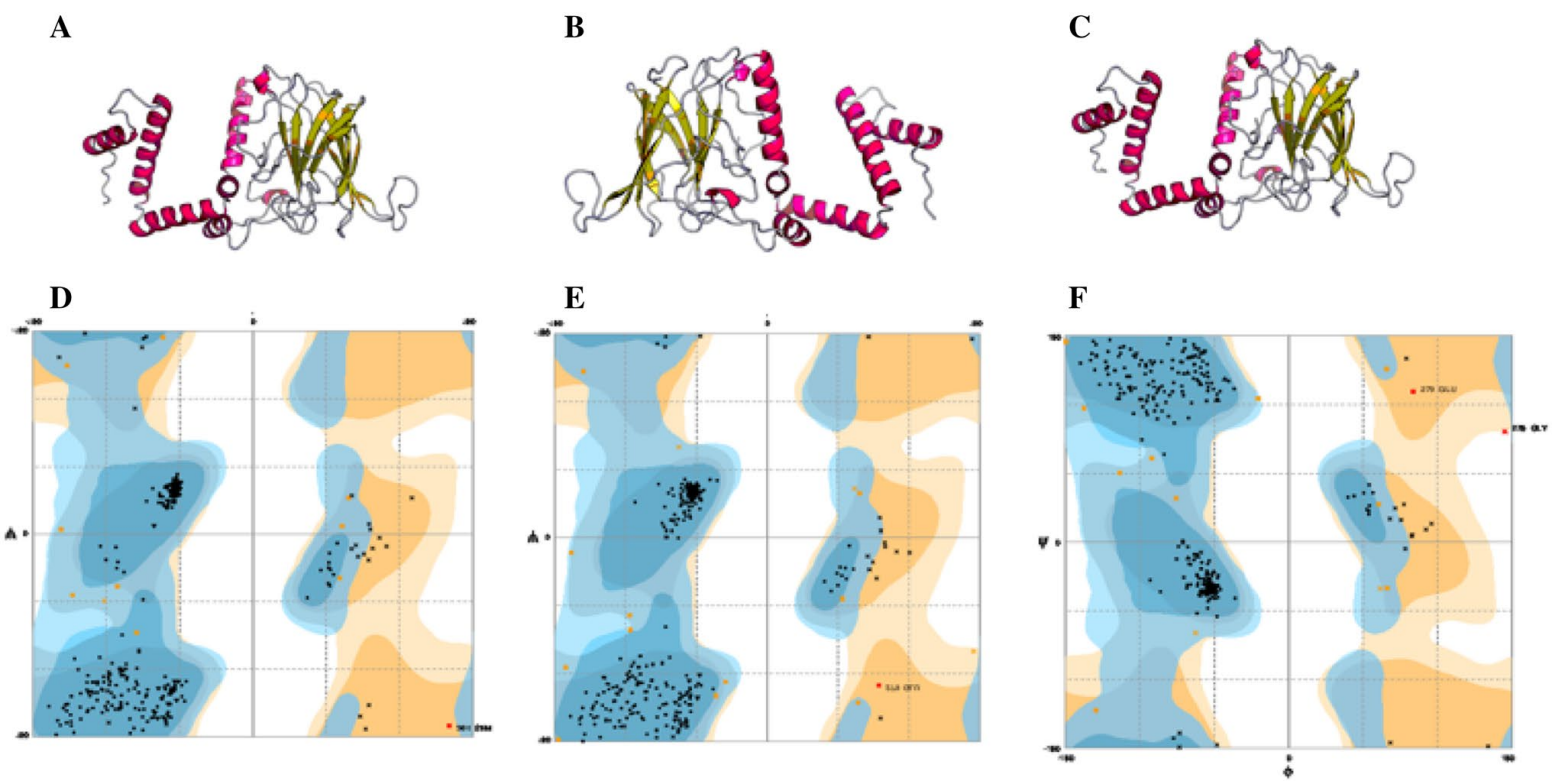
**a** 3D structure of catechol 1, 2-dioxygenase (*catA*) protein of AWD5 generated by RaptorX, with **b** *K. oxyto*ca and **c** *K. pneumoniae,* respectively. **d**, **e**, **f**, Ramachandran plot of *catA* with respect to the above structure, where more than 90% of amino acid residues are distributed in the favored region

The predicted 3D modeled structure of *catA* was analyzed using RaptorX. Ramachandran plots were generated by Rampage using pdbSum and the result indicates that the number of amino acid residues in favored region is 295 (96.4%), and that the number of amino acid residues in allowed region is 10 (3.3%). Comparison of amino acid sequence alignment of the gene with other two bacteria showed 89.0 and 92.9% similarity with *K. oxytoca* and *K. pneumoniae,* respectively (Fig. 8).

### Genetic elements for degradation of benzoate and fluorobenzoates

Benzoate degradation by *K. pneumoniae* AWD5 undergoes through β-ketoadipate (3-oxoadipate) pathway. The degradation of benzoate by AWD5 is initiated by *benA-*coded enzyme that transforms benzoate to *cis*-1,2-dihydroxycyclohexa-3,5 diene 1 carboxylate, which is further catalyzed by *benD* to catechol as early intermediates. The catechol is oxidized via the ortho-ring cleavage mechanism, which serves as substrate for the dioxygenase enzyme (*catA*) to cleave the aromatic ring between hydroxyl groups leading to 3-oxoadipate, which is then converted to succinyl CoA and followed a series of reaction to citrate cycle. In the proposed catechol branch pathway, where catechol generated from benzoate was converted into β-ketoadipate through the action of the *ben* gene products. The protocatechuate branch, encoded by *pca* genes, converts the protocatechuate derived from 4-hydroxybenzoate into β-ketoadipate (Pantoja et al. 2008). Bioinformatics analysis predicts that *benA* (benzoate 1, 2-dioxygenase α-subunit), *benB* (benzoate 1, 2-dioxygenase β-subunit), and *benD* (a diol dehydrogenase) genes were responsible for benzoate degradation in AWD5.

**Fig. 9:**
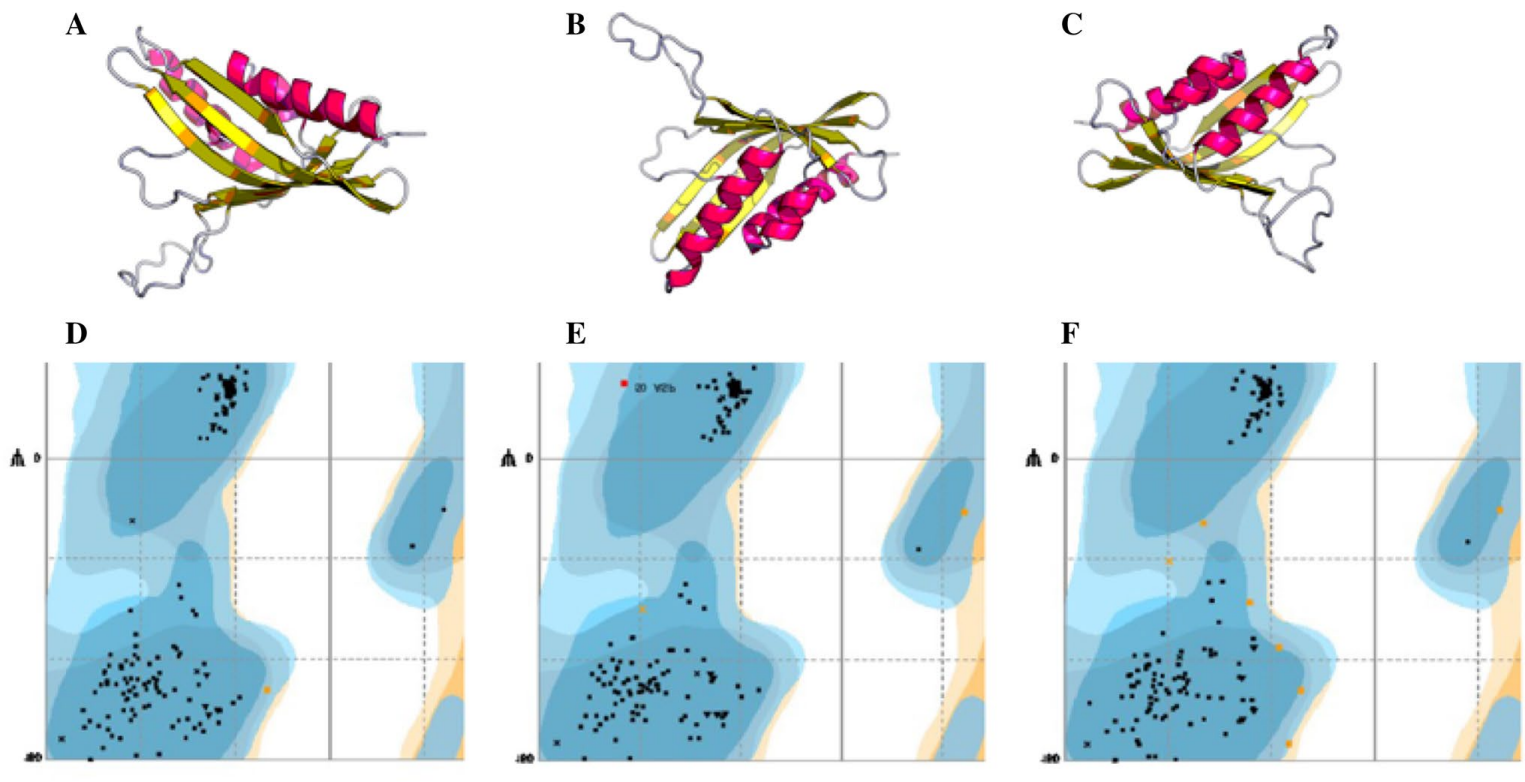
**a** 3D structure of benzoate 1, 2-dioxygenase β-subunit (*benB*) protein of AWD5 generated by RaptorX, with **b** *Enterobacter aerogenes* and **c** *K. pneumoniae,* respectively. **d**, **e**, **f**, Ramachandran plot of *benB* with respect to the above structure, where more than 95% of amino acid residues are distributed in the favored region

The 3D structure benzoate 1, 2-dioxygenase β-subunit of AWD5 was tested by RaptorX. The number of amino acid residues in favored region was found to be 156 (98.1%) and number of amino acid residues in allowed region is 3 (1.9%) as generated by Ramachandran plot. Comparison of amino acid sequence alignment of the gene with other two bacterial genes showed 96.9 and 98.8% similarity with *Enterobacter aerogenes* and *K. pneumoniae,* respectively (Fig. 9).

2- and 3-fluorobenzoate was shown to be degraded by *K. pneumoniae* AWD5 by benzoate catabolic enzymes via 3-oxoadipate pathway with the formation of 3-fluorocatechol and 4-fluorocatechol as intermediate, respectively. Through ortho-cleavage pathway, catechol 1, 2-dioxygenase catalyzed the 3-fluorocatechol intermediate yielding 2-fluoro-cis, cis-muconate, and 5-fluoromuconolactone, whereas 4-fluorocatechol was metabolized into 3-fluoro-cis, cis-muconate, and 4-fluoromuconolactone. These products were further transformed into maleylacetate by deflurorination by the action of carboxymethylenbutenolidase. During fluorobenzoate degradation, 4-fluorocatechol and catechol were formed as intermediates through aerobic metabolism of fluorobenzene (Fig. 7). The AWD5 strain also shows the ability to degrade 4-fluorobenzoate in the similar pathway where 3-fluorobenzoate degradation occured.

### Genetic elements for degradation of 3-hydroxyphenyl propionic acid

**Fig. 10:**
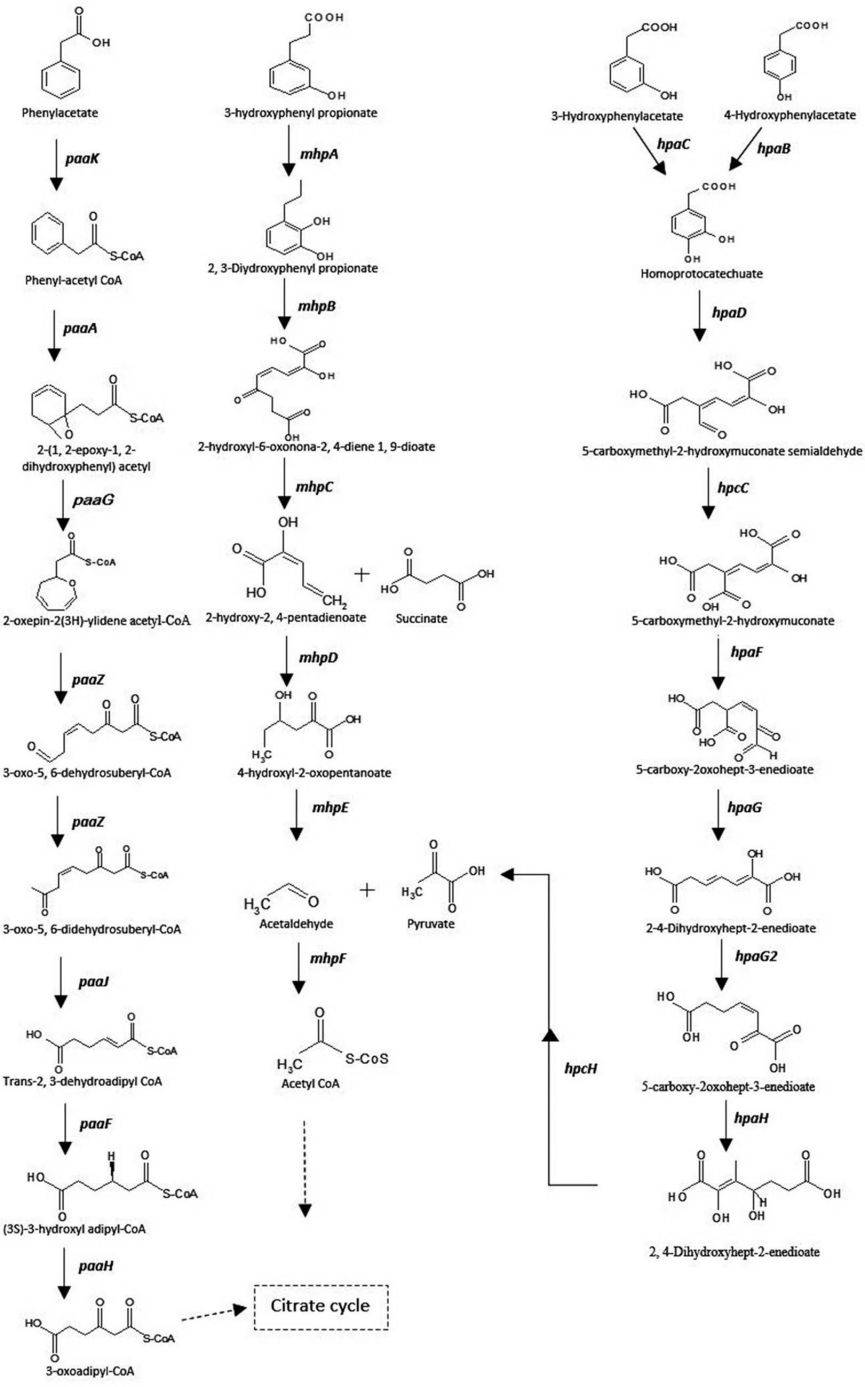
Phenyl acetic acid, 3-hydroxyphenyl propionate, and 3-, 4-hydroxyphenylacetate degradation pathway by *K. pneumoniae* AWD5 with their intermediates and responsible genes

**Fig. 11:**
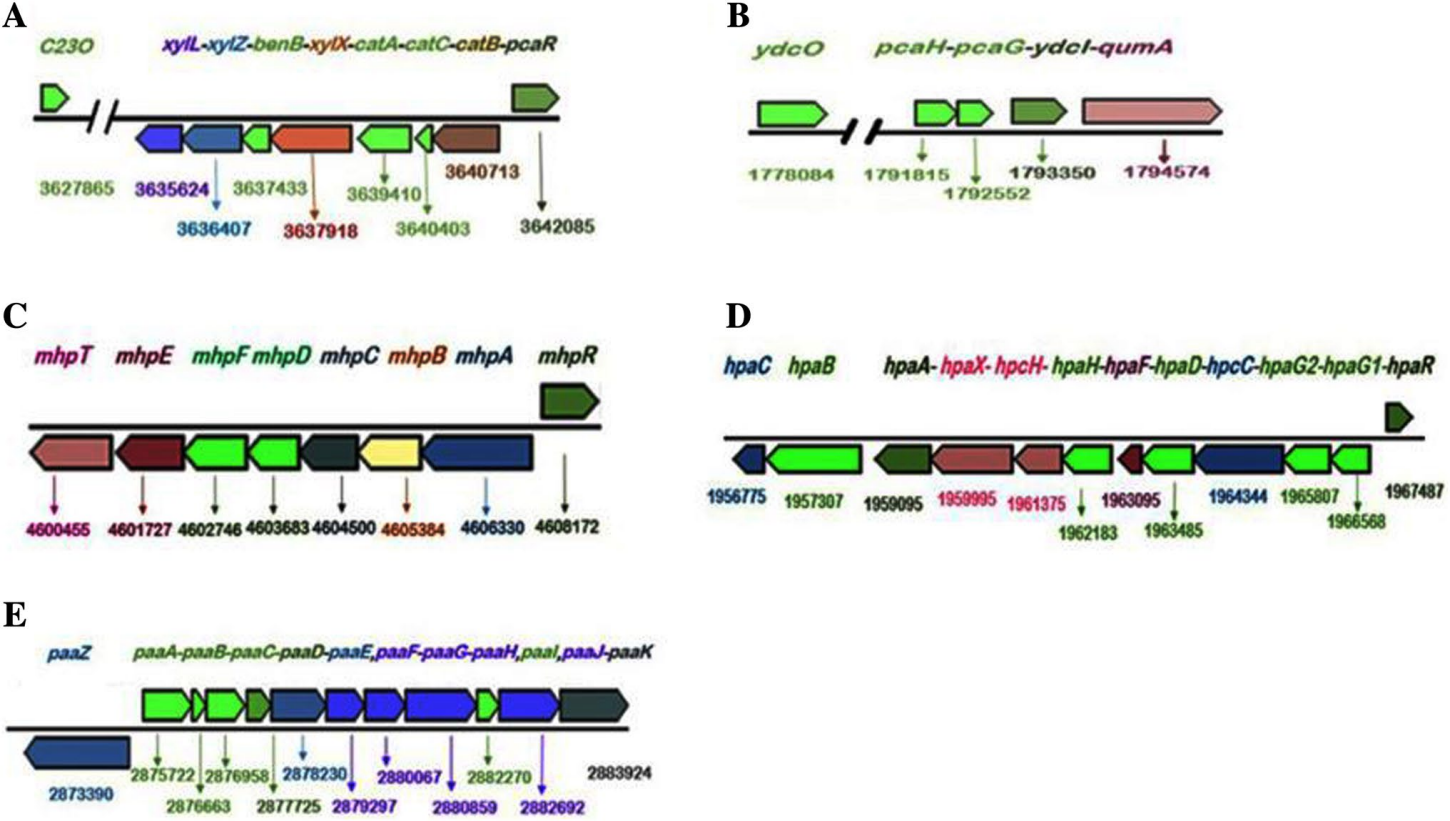
Arrangements of gene clusters in genome of *K. pneumoniae* AWD5 identified for degradation of aromatic compounds. **a** dioxygenase genes for aromatic hydrocarbons; **b** *pcaGH* gene set for protocatechuate breakdown; **c** *mhp* genes for 3-hydroxyphenyl propionic acid; **d** *hpa* gene cluster for 3-hydroxyphenylacetate cleavage; **e** *paa* genes for phenyl acetic acid degradation. The name and functions of genes are described in the text

Genome analysis revealed the presence of 3-hydroxyphenyl propionic acid (3-HPP) degradation pathway in AWD5, encoded by the *mhp* genes cluster. These eight *mhp* genes (*mhpTEFDCBAR*) were known to transforms 3-hydroxyphenyl propionic acid (3-HPP) to TCA cycle intermediates. The aerobic degradation of 3-hydroxyphenylpropionate (3-HPP) was initiated by a monooxygenase, 3-hydroxyphenyl propionate hydroxylase (*mhpA)* forming 3-dihydroxyphenyl propionate as central intermediate, and it is further degraded through meta-cleavage hydrolytic pathway. The intermediate was transformed to 2-hydroxy-6-oxonona-2, 4-diene-1, 9-diote catalyzed by 2, 3-dihydroxyphenyl propionate 1, 2-dioxygenase (*mhpB*). The product was hydroxylated by 2-hydroxy-6-ketonona-2, 4-dienedioate hydrolase (*mhpC*) followed by a series of reactions catalyzed by 2-keto-4-pentanoate hydratase (*mhpD*), 4-hydroxy-2-oxovalerate aldolase (*mhpE*), and acetaldehyde dehydrogenase (*mhpF*). The cluster *mhpCDFE* code for the hydrolytic *meta*-cleavage to give acetyl co-A as the final product (Fig. 10). *K. pneumoniae* AWD5 also contained *mhpR* as a transcriptional regulatory gene, located adjacent to *mhpA* in the opposite orientation and *mhpT,* which encoded MFS transporter protein (3-hydroxyphenyl propionic acid transporter). The *mhp* gene cluster arrangement is given (Fig. 11c).

### Genetic elements for degradation of 3- and 4-hydroxyphenylacetate

Gene cluster for 3-hydroxyphenylacetate (3-HPA) and 4-hydroxyphenylacetate (4-HPA) cleavage pathways were identified in *K. pneumoniae* AWD5 genome. 3- and 4-hydroxyphenylacetate were hydroxylated to give intermediate compound homoprotocatechuate, catalyzed by 4-hydroxyphenyl acetate 3-monooxygenase reductase component encoded by *hpaC* and 4-hydroxyphenylacetate 3-monooxygenase encoded by *hpaB,* respectively. The pathway was followed by the formation of 5-carboxymethyl-2-hydroxymuconate semialdehyde (CHMS) subsequently catalyzed by *hpaD* and *hpcC.* The gene cluster *hpaBCDFG* and *hpcCH,* with a regulatory gene *hpaR* that was involved in the catalytic pathway of hydroxyphenylacetate, were also identified in the genome. The product was further changed into 5-carboxy-2-oxohept 3-enedioate by 5-carboxymethyl-2-hydroxy muconate delta isomerase (*hpaF*) which ultimately leads to the formation of TCA cycle intermediate, pyruvate, and succinate semialdehyde catalyzed by 4-hydroxy-2-oxo-heptane-1, 7 dioate aldolase (*hpcH*), and succinate semialdehyde dehydrogenase (*gabD*) catalyzed succinate semialdehyde into succinate in AWD5. *K. pneumoniae* AWD5 metabolized HPA through meta-cleavage pathway (Fig. 10). The organization of *hpa* gene cluster in AWD5 is given in Fig. 11d.

### Genetic elements for degradation of phenylacetate degradation

Phenylacetate is a key intermediate in the degradation of various environmental pollutants (Teufel et al. 2011). Phenylacetate catabolic gene cluster (*paaZ* and *paaABCDEFGHIJK*) was organized as single operon-encoding enzymes in *K. pneumoniae* AWD5 (Fig. 11e). The first step in degradation of phenylacetate pathway in *K. pneumoniae* AWD5 was the activation of phenylacetate to phenylacetyl-CoA by a phenylacetate-CoA ligase (*paaK*). The product was catalyzed to 2-(1, 2-epoxy-1, 2-dihydrophenyl) acetyl CoA by ring 1, 2-phenylacetyl-CoA epoxidase (*paaA*). The non-aromatic epoxide was further isomerized by phenyl acetate degradation probable enoyl-CoA hydratase *paaB* (*paaG*) to 2-oxepin-2(3H)-ylideneacetyl-CoA and the ring was cleaved and subsequently catalyzed by *paaZ*, *paaJ*, *paaF*, *paaH*, and *paaJ* leading to the formation of citric acid cycle intermediates succinyl CoA. Its degradation pathway is given (Fig. 10).

### Genomic islands (GIs) in *K. pneumoniae* AWD5

A number of genomic islands were found to be very less in genome of *K. pneumoniae* AWD5 as only six regions across the AWD5 genome were identified, comprising a total of 100 genes. There were no curated virulence factors, homologs of virulence factors, curated resistance genes, homolog of resistance genes, and pathogen-associated gene in GIs of AWD5. These GIs consisted operons for fimbrin like proteins (*fimEICDFGH*), phage shock protein (*pspABCD*), with the *psp* operon transcriptional activator *pspF,* and outer membrane proteins.

### Plant growth promoting attributes of *K. pneumoniae* AWD5 and its genetic elements in genome

AWD5 produce 14.75 µg/ml of IAA in pyrene amended nutrient broth medium, whereas it was recorded to produce 95.31 µg/ml in glucose amended medium. The plant growth promoting attributes of AWD5 are given in Table 4. IAA biosynthesis genes are identified in the AWD5 genome. The genome has *iaaH* genes encoding indole 3-acetamide hydrolase which is responsible for converting indole-3-acetamide (IAM) to IAA in Indole-3-acetamide pathway. In addition, indole-3-pyruvate decarboxylase (*ipdC*) was identified which plays key role in IAA synthesis via the intermediate indole-3-pyruvate. *ipdC* was induced by transcriptional regulatory protein *tyrR*.

**Table 4:** Plant growth promoting attributes

PGP attributes	In the presence of glucose	In the presence of pyrene
IAA (µg/ml)	95.31 ± 1.54a	14.75 ± 0.35a
Phosphate solubilization (ng/ml)	150.14 ± 3.03	198.28 ± 2.43
Siderophore production (%)	58.81 ± 2.68a	13.56 ± 2.24a
ACC deaminase activity (mM α ketobutyrate/mg)	5.39 ± 0.028a	0.118 ± 0.011a

± Standard error; a-significance (*p* < 0.05)

**Fig. 12:**
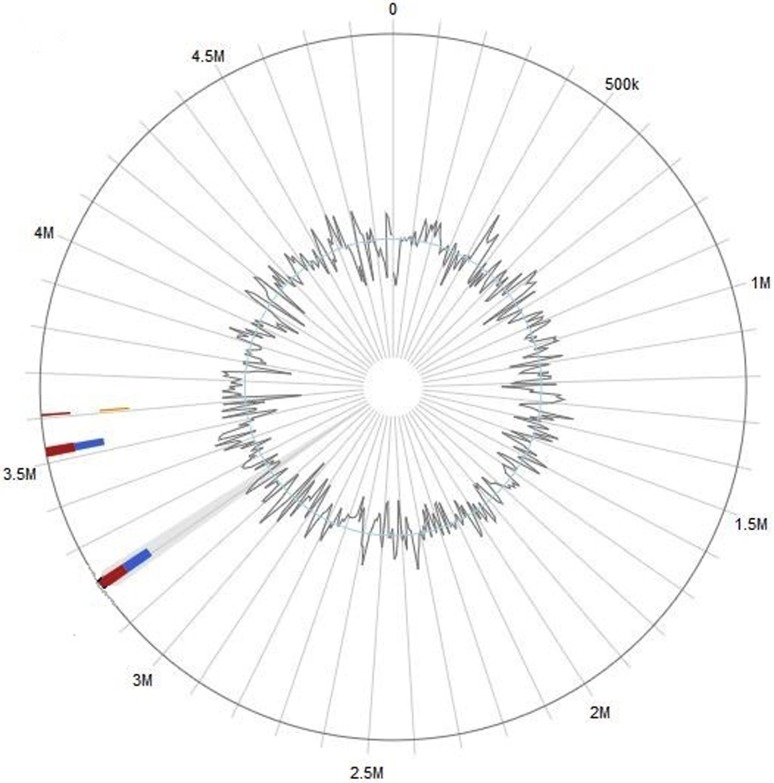
Genomic island prediction of *K. pneumoniae* AWD5

The phosphate solubilizing activity was estimated to be 150.14 and 198.28 ng/ml in glucose and pyrene amended National Botanical Research Institute Plant growth (NBRIP), respectively, medium. Genes involved in mineral phosphate solubilization were encoded from the AWD5 genome. Mineral phosphate solubilization is related to production of gluconic acid (GA). Gluconic acid biosynthesis is carried out by glucose dehydrogenase (GDH) and co-factor pyrrolo-quinolone quinine (PQQ) (Rodriguez et al. 2006). AWD5 genome has genes encoding glucose dehydrogenase activity and PQQ genes including *pqqBCDEF*. Moreover, inorganic phosphate uptake transport systems were also present, which is known to promote the uptake of phosphate by low affinity phosphate transport system *pitA* and high-affinity transport system *pstBACS* (Fig. 12).

Similarly, the amount of siderophore production activity was significantly higher in glucose amended medium (58.81%) than pyrene amended medium (13.56%). *K. pneumoniae* AWD5 was found to release siderophore in iron-deficient conditions. Multiple genes were identified in the genome of *K. pneumoniae* AWD5 for siderophore production and its transport. Biosynthesis of siderophore genes was encoded by *entABCECF* and export of this siderophore out of the cell was encoded by *entS*. The *fep*- gene cluster that code for transport of enterobactin-type siderophore were identified, like *fepA* that encode—the outer membrane receptor, *fepC*—ferric enterobactin transport ATP-binding protein, *fepG* and *fepD*—transport system permease protein, and *fepB*—periplasmic binding protein along with catecholate type of siderophore receptors encoded by *fiu* and *ybiL*. Ferric enterobactin processed via specific pathway depends on FES activity, making iron available for metabolic use encoded by the gene *fes*. The genes for siderophore receptors including *tonB*-dependent receptors (*yncD*, *fhuAI, and pfeA*) and iron uptake system permease protein (*feuC*), iron (3 +)-hydroxymate import system permease protein (*fhuB*), siderophore transport system ATP-binding protein (*yusA*), ferric aerobactin receptor (*iutA*), ferrioxamine receptor (*foxA*), ferrichrome receptor (*feuA*), and ferric uptake regulation protein (*fur*) were present.

**Fig. 13:**
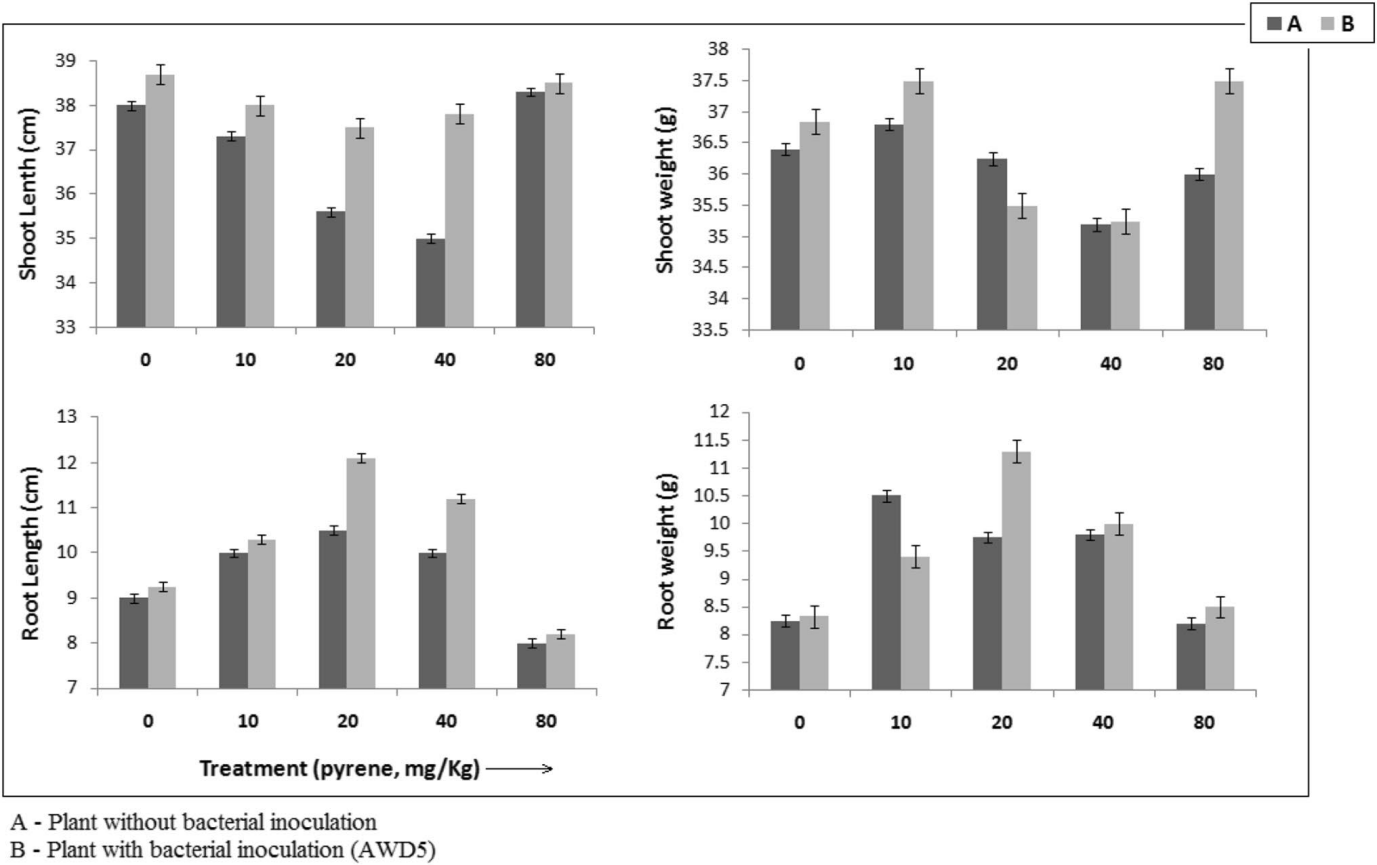
Effect of AWD5 inoculation on the growth of *J. curcas* at different concentration of pyrene in soil

AWD5 produces 5.3 mM α-ketobutyrate/mg ACC deaminase activity which was significantly higher in pyrene amended DF salt medium (0.118 mM α ketobutyrate/mg). AWD5 improved the growth of *J. curcas* in pyrene-contaminated soil (Fig. 13). The growth parameters for roots were better than control in 20 mg/kg pyrene, in AWD5-augmented soil, where 13.2 and 13.7% increase in root length and weight was recorded, respectively. Furthermore, 7.4% increase in the shoot length was observed in the presence of 40 mg/kg of pyrene in AWD5 augmented soil, as compared to control.

### Genetic elements for metal resistance in *K. pneumoniae* AWD5

*Klebsiella pneumoniae* AWD5 carries genes for transport of elements and resistance of heavy metal. A part of cation efflux system that mediates resistance to copper and silver (*cusABFCRS*) were found to be present in the strain. The *Cus* determinant of the strain AWD5 was categorized as structural (*CusCFBA*) and the other regulatory (*CusRS*) operon.

Ni is an essential component of several metalloenzymes involved in energy and nitrogen metabolism (Mulrooney and Hausinger 2003). AWD5 encoded nickel uptake genes cluster *nikABCDE* where *nikA* encoded nickel-binding periplasmic protein, *nikB, nikC* encoded nickel transport system permease protein, *nikD*, *nikE* encoded for nickel import ATP-binding protein, *nikR* coded for nickel-responsive regulator.

Different genes that are involved in transport, processing, and synthesis of molybdate and molybdopterin are identified in *K. pneumoniae* AWD5 genome. The molybdate transport system of *K. pneumoniae AWD5* was encoded by *modABC* genes and transcriptional regulator was encoded by *modE*. Molybdenum co-factor biosynthesis protein was encoded by *moaA*, *moaB*, *moaC*, and molybdopterin synthase sulfur carrier subunit encoded by *moaD*. Molybdopterin molybdenum transferase protein was encoded by *moeA*, sulfur carrier protein moaD adenyltransferase was coded by *moeB,* and catalytic subunit was encoded by *moaE*. Molybdopterin guanine dinucleotide biosynthesis protein was coded by *mobA* and *mobB*. *CbiMNQO* were believed to mediate cobalt uptake in prokaryotes (Eitinger et al. 2005). Cobalt import ATP-binding protein was coded by *cbiO* and cobalt transport proteins were encoded *cbiQ*, *cbiN*, and *cbiM.* Cobalt-precorrin-3B C(17)-methyltransferase *cbiL*, *cbiH*, sirohydrochlorin cobalt chelatase *cbiK*, cobalt-precorrin-6A reductase *cbiJ*, protein *cbiG*, cobalt-precorrin-4 C(11)-methyltransferase *cbiF*, probable cobalprecorrin-6Y C(15)-methyltransferase (decarboxylating) *cbiT*, *cbiE*, putative cobalt-precorrin-8X methyltransferase *cbiC* were found in the genome of AWD5.

## Discussion

The draft genome sequence of *K. pneumoniae* AWD5 was sequenced to study the mechanism underlying degradation of aromatic compounds. The complete and draft genome sequences of environmental isolates *K. pneumoniae* J1, *K. pneumoniae* KP-1 and clinical isolates *K. pneumoniae* KP617, *K. pneumoniae* U25 have been reported (Lee et al. 2013; Kwon et al. 2016; Pang et al. 2016; Rafiq et al. 2016). Their statistics also clearly suggested that there is intra-species variation in coding and RNA elements in *K. pneumoniae*. The genome data of AWD5 strain supported and extended various laboratory observations in the plant growth promotion attributes.

From the comparative analyses, it is observed that selected *K. pneumoniae* genomes of environmental as well as clinical origins were highly similar to that of strain AWD5, and in addition, there were no specific arrangement in regions of variations between all the genomes. Hydrocarbon degrading genes were found to be conserved in *K. pneumoniae*. Interestingly, the genome of ATCC BAA-2146 (clinical, reference strain) completely lacked hydroxy phenyl propionate degradation operon (*mhpTBCADFE*), which was otherwise present in environmental isolates AWD5 and KP-1. However, AWD5 did not have any specific infection-related gene, and only non-specific genes which had been suggestive of indirect roles in disease were identified, such as putative protease, succinate dehydrogenase subunit alpha, chaperonin Gro EL, amino acid-binding domain sensor hybrid histidine kinase, adenylate cyclase, and two component transcriptional regulator (LuxR family). ATCC BAA-2146 and KP-1 was found to have infection-related genes like *ureC* (epithelial cell signaling) gene and also oligopeptidase B, which were absent in AWD5. Previously, Kwon et al. (2016) had also reported *K. pneumoniae* PittNDM01 without any unique virulence factor which was isolated from urine sample of a patient. Similarly, *K. pneumoniae* KP617 which was also a clinical isolate had been reported to harbor 117 virulence genes, but did not possess any unique virulence factors (Kwon et al. 2016). Though other strains like NUHL24835 and ATCC BAA-2146 were reported to have three and seven unique virulence factors, respectively. These clinical strains (ATCC BAA-2146, PittNDM01, NUHL24835, KP617) were reported to encode the NDM-1 metallo-β-lactamase, which was absent in AWD5.

*Klebsiella pneumoniae* AWD5 was isolated from oil-contaminated soil and, therefore, has potential for implementation in oilfield bioremediation. Numerous genes associated with aromatic compounds degradation were identified. The gene sets available in the genome indicate that *K. pneumoniae* AWD5 metabolize hydrocarbons using both, ortho- and meta-cleavage pathways. The in silico analysis and prediction of biochemical metabolism of PAH by *K. pneumonia* AWD5 favored degradation in aerobic condition via oxygen-mediated metabolism. In addition, *K. pneumoniae* AWD5 genome also possesses multiple dioxygenase genes, and hence, it has ability to putatively undergo a complete β-ketoadipate pathway through catechol of ortho-cleavage pathway for further degradation of the ring cleavage products to TCA cycle intermediates. This was further confirmed by quantitative estimation of C-1,2 D and C-2,3 D released by AWD5 in extracellular medium. This pathway was considered to be one of the key routes for the degradation of aromatic compounds. *K. pneumoniae* 342 strain had been suggested to metabolize hydrocarbons in the similar pathway (Fouts et al. 2008). Members of the genus *Pseudomonas*, *Rhodococcus, Serratia, Flavimonas, Klebsiella*, *Pantoeba, Burkholderia, Serratia*, and *Microbacterium* spp. had been reported to utilize catechol by ortho- cleavage pathway in the presence of aromatic compounds (Song 2009). This indicates that AWD5 has a broad potential for the degradation of aromatic compounds.

*Klebsiella pneumoniae* AWD5 has the ability to degrade benzoate putatively through *ortho*-cleavage of β-ketoadipate pathway forming cis–cis muconic acid (ccMA) as an intermediate of this pathway leading to tricarboxylic acid cycle (TCA) intermediates. Similar pathway has been reported in *P. putida*, *A. evansii*, *C. necator* strains, *Acinetobacter* sp. KS-1 (Feist and Hegeman 1969: Kim et al. 2006a, b; Pantoja et al. 2008). 2- and 3-fluorobenzoate degradation by *K. pneumoniae* AWD5 formed 3- and 4-fluorocatechol as early intermediate, respectively. During fluorobenzoate degradation, 4-fluorocatechol and catechol were formed as intermediates by *Rhizobiales* sp. strain F11 through aerobic metabolism of fluorobenzene. Conversion of a fluorinated compound to catechol and a fluorinated catechol had been described for the degradation of 2-fluorobenzoate by *Pseudomonas* sp. strain B13 and strain FLB300. The metabolism of fluorocatechol in *K. pneumoniae* AWD5 progresses through ortho-cleavage pathway, which is also a key step of fluorobenzoate degradation in numerous bacterial strains cleaved by catechol 1, 2-dioxygenase that yields 3-fluoro-*cis*, *cis*-muconate (Carvalho et al. 2006) and then channeled into 3-oxoadipate pathway (Harper and Blakley 1971; Schreiber et al. 1980, Engesser et al. 1990).

The gene clusters of 3-hydroxyphenyl propionate (3-HPP) catabolic genes were encoded by *mhp* gene clusters in *K. pneumoniae* AWD5 which resembles with previously reported *E*. *coli*, *Comamonas testosteroni* TA441, and also in *K. pneumoniae* (Gibello et al. 1997; Arai et al. 1999). This *mhp* cluster contained catabolic genes for the catabolism of 3HPP (Xu et al. 2013). The genes involved in 3-HPP catabolism including the *mhpRABCDFET* operon were identified in *Klebsiella* sp. DH5 (Liu et al. 2016). However, in *R*. *globerulus* PWD1, 3-HPP degradation was encoded with different gene organization (Barnes et al. 1997). The organisms utilized the common pathway of 3-HPP degradation through meta-cleavage putative pathway to form TCA cycle intermediates as in *K. pneumoniae* AWD5.

Homoprotocatechuate degradation pathway had been described as a central route for the catabolism of aromatic amino acids in *K. pneumoniae, P. putida*, and *E. coli* (Mendez et al. 2011). Analysis of the HPC catabolic pathways genes of *E. coli* C was located in two operons, *hpcBCDEF* and *hpcGH* with a regulatory gene, *hpcR* (Jenkins and Cooper 1988). Martín et al. (1991)reported that *K. pneumoniae* metabolized 4-HPA through a *meta*-cleavage pathway with 3,4-dihydroxyphenylacetic acid (3,4-DHPA) as the dihydroxylated intermediate which was catalyzed by 3,4-Dihydroxyphenyl acetate 2,3-dioxygenase (*hpcB*). Succinate and pyruvate were formed as the final products. However, in the present study, *K. pneumoniae* AWD5 was found to catalyze 3-HPA and 4-HPA by *hpaD,* and was found to proceed in the similar putative degradation pathway.

Phenylacetic acid catabolic genes had been found in *E. coli*, *Pseudomonas putida, Azoarcus evansii,* and *Rhodococcus* spp. (Ferransdez et al. 2000; Olivera et al. 1998; Mohamed et al. 2002; Navarro-Llorens et al. 2005) catalyzing the degradation in four steps via phenylacetyl-coenzyme A (CoA). Phenylacetate metabolism has been reported in a variety of bacteria with the responsible genes that ultimately convert to succinyl CoA and acetyl CoA (Luengo et al. 2001; Teufel et al. 2011). However, in *K. pneumoniae* AWD5 putative pathway for phenylacetate degradation, the genes were found to be encoded by 11 gene clusters of *paa*. The paa gene cluster organization was described to be conserved in *R. jostii* RHA1 and *R. opacus* R7 genomes (Orro et al. 2015). The genes *paaA*, *paaB*, *paaC*, *paaD,* and *paaE* involved in ring hydroxylating, whereas *paaG, paaZ,* and *paaJ* genes were involved in opening of aromatic ring, which was followed by degradation through β-oxidation similar pathway by *paaF*, *paaH,* and *paaJ* (Ismail et al. 2003; Nogales et al. 2007).

There were only few GIs present in genome of AWD5, which indicated towards a stable genome. It may also be indicative of less vulnerable to genetic transfer. As a matter of fact, other *K. pneumoniae* genomes had been reported to have more GIs as they possessed numerous laterally transferred genes and antibiotic resistance GIs. In the genome of an environmental isolate, *K. pneumoniae* KP-1, 70 GIs were predicted, out of which two belong to antibiotic resistance GIs. Similarly, *K. pneumoniae* Kp342 had 429 GIs with eight antibiotic resistance GIs. While clinical isolate *K. pneumoniae* ATCC BAA 2146 had 116 GIs out of these, eights GIs were pathogen-associated and nine were resistant GIs (Bertelli et al. 2017). Interestingly, the GIs of environmental isolates of *K. pneumoniae*, including AWD5, were not having virulence associated genes, yet they were present in clinical isolates.

*Klebsiella pneumoniae* AWD5 has elements for several plant growth promoting attributes, which suggested the role to promote plant growth being a soil isolate. IAA is a plant growth regulator, which is required for plant growth and development. Soil bacteria synthesized IAA using tryptophan by one or more pathways. AWD5 genome has genes for two different pathways, i.e., indole 3-acetamide and indole 3-pyruvate for production of IAA. The role of *ipdC* gene in IAA production had been experimentally confirmed in *E. cloacae, A. brasilense,* and *P. agglomerans* (Patten et al. 2013). In addition, AWD5 has phosphate solubilization ability, which is useful in providing soluble phosphates to plants. AWD5 was found to have gluconic acid forming ability, which was known to solubilize mineral phosphate in soil. In addition, AWD5 has *pqqBCDEF* locus, which directed the synthesis of PQQ, a co-factor of glucose dehydrogenase holoenzyme (GDH) (Meulenberg et al. 1992). Bhardwaj et al. (2017) reported that *K. pneumoniae* VRE36 produced 45 µg/ml of IAA and estimated 17.4 µg/ml release of available phosphate in NBRIP medium.

Siderophores are iron chelating compounds secreted by microorganisms (Neilands 1995). The rhizobacteria have the ability to produce siderophore increase plant growth in soil (Cattelan et al. 1999). Soil bacteria assimilate Iron (III) by excreting siderophores that selectively bind iron (III) to form complexes. These complexes are taken and iron thus acquired can be utilized by dissimilatory iron (III)-reducing bacteria. It is coupled with iron (III) reduction to oxidative degradation of organics, as well as iron (III)-solubilization (Kamnev et al. 1999). *K. pneumoniae* AWD5 synthesized enterobactin type of siderophore. It is the main siderophore produced by *Klebsiella* sp. Besides, it has been observed that *Klebsiella* isolates synthesized aerobactin rarely, suggesting that aerobactin is not a principal mechanism of iron acquisition in *Klebsiella* spp. (Podschun et al. 1992). However, siderophore was also reported to be virulent factor in many Gram-negative bacteria (Holden et al. 2016). Some scientists proposed that siderophores assisted infection by promoting bacterial growth, such as enterochelin (enterobactin) and aerobactin in *Klebsiella* spp. (Blum 2016). Though Podschun et al. (1992) reported that the role of enterobactin in infection was unclear and these factors had no covalent relation for virulence in *K. pneumoniae*.

The strain AWD5 showed C-1,2D and C-2,3D enzyme activity in pyrene, benzo-a-pyrene, and chrysene amended medium. *K. pneumoniae* had been reported for its capability to degrade polyaromatic hydrocarbons (Ping 2014). In this study, higher activity of both the enzymes was observed in pyrene and benzo(a)pyrene than chrysene amended medium and also from the comparison of percentile degradation of each PAH. The activity of C-2,3D was reported in *Pseudomonas* strains to be detected only in the presence of suitable inducers (catechol, benzoate, and salicylate), while basal activity of C-1,2D was detected even in the absence of inducers, and increased twofold to that of control in presence of chrysene and benzanthracene (Cenci et al. 1999). Kotoky et al. (2017) suggested that activity of C-1,2D and C-2,3D increases in the presence of benzo(a)pyrene, which is similar to our observations with pyrene.

In general, ACC deaminase exhibits optimum activity at a pH close to 8; however, this might vary depending on the microbial species (Jacobson et al. 1994; Minami et al. 1998; Jia et al. 1999; Hontzeas et al. 2004). Plants inoculated with ACC deaminase bacteria that express bacterial ACC deaminase genes regulate their ethylene levels and, therefore, contribute to a more extensive root system. Such proliferation of roots in contaminated soil leads to enhance uptake of heavy metals or rhizodegradation of xenobiotics (Arshad et al. 2007). AWD5 was found to exert beneficial growth on plant, thereby degrading contaminants in the pyrene-contaminated soil. The root growth was improved with application of AWD5, as compared to control, which might be due to its ACC deaminase production ability. It had been reported that *K. pneumoniae* strains were found to enhance the plant growth, seed germination rate in maize, wheat, sugarcane, etc. (Sachdev et al. 2009; Kuan et al. 2016; Bhardwaj et al. 2017).

Transcription initiation of *cusCFBA* is dependent on the concentration of copper and silver (Munson et al., 2000). The *CusRS* operon encodes a histidine kinase, *CusS* located in the inner cell membrane, and *CusR* is a transcriptional regulatory protein present in cytoplasm. *CusA* and *CusB* are essential for copper resistance, and *CusC and CusF* are required for full resistance (Franke et al. 2003). Molybdenum is an essential trace element required for the enzyme activity in the form of a molybdenum co-factor which was found in bacteria, plants, and animals (Rajagopalan and Johnson 1992). Molybdo-enzymes (except dinitrogenase) contain a unique form of molybdopterin-nucleotide as the co-factor. Molybdopterin has a terminally phosphorylated, four-carbon alkyl side chain with a dithiolene group, and two sulfur atoms of which is ligand to the molybdenum (Rajagopalan and Johnson 1992). The *moa* and *moe* loci are required for molybdopterin biosynthesis (Rivers et al. 1993). The molybdate transport systems in *E. coli*, *A. vinelandii* and *R. capsulatus* were similarly constructed (Luque et al. 1993; Wang et al. 1993). Cobalt is a trace element which is required for various biological processes and it is also a component of vitamin B12 (Zhang et al. 2009). Nickel uptake by the periplasmic binding protein is encoded by *nikABCDE* (Wu et al. 1991). Microorganisms incorporate this metal ion into metabolic reactions of hydrogen metabolism, ureolysis, methane biogenesis, and acetogenesis (Hausinger 1987). The presence of gene blocks for metals like Fe, Ni, Co, and Mo confirmed the ability of AWD5 to acquire these, in limiting environment and also have competitive survival ability in Cu/Ag contaminated soil. Previously, adsorption abilities for heavy metals have been reported in an environmental isolate *K. pneumoniae* J1 (Pang et al. 2016). Though this property was not confined to environmental isolates, as clinical isolate like *K. pneumoniae* MGH78578 (Seo et al. 2012) has also been reported to contain Ni and Co transport genes.

Therefore, the genome of *K. pneumoniae* AWD5 was found to be very unique, as being a soil isolate. Overall, there were no considerable variations from genomes of clinical isolates, but there was no major disease-related genetic elements found in the genome of AWD5. In addition, the genome has features to provide versatility to the isolates, as it has ability to degrade wide spectrum of hydrocarbons due to the presence of hydrocarbon degrading operons like *paaZABCDEFGHIJK, mhpTBCADFE, hpaCBXFDG*_*1*_*G*_*2*_*R, hpcCH, benCB, catACB,* and *pcaDGHIJ*. Elements for heavy metal resistance were also present in addition to plant growth promoting attributes such as IAA, siderophores, and phosphate solubilization. The genome has very low number of GIs with no virulent-associated genes.

## Conclusion

The genome study of *K. pneumoniae* AWD5 confirmed that indigenous bacterial strain isolated from contaminated site is capable of degrading aromatic compounds in soil. Comparative genomics approach suggested a significant similarity among *K. pneumoniae* genomes of clinical and non-clinical origins, when compared with AWD5. A significant homology of the genes involved in hydrocarbon degradation was observed through the comparative analysis of the genome between different genomes of *K. pneumoniae*. Actually, overlapping functional characteristics like biodegradation and virulence were identified in these genomes, with considerable synteny, AWD5 being exception where no unique gene for virulence was identified. From the genome analysis, it was apparent that AWD5 encodes several oxygenases that lead to cleavage of the aromatic ring by accepting activated molecular oxygen. In fact, the strain was found to be versatile and degrade wide range of complex hydrocarbon containing multiple genes of aromatic and polyaromatic hydrocarbons degrading genes. Putative degradation pathway of polyaromatic hydrocarbon, benzoate, phenylacetate, 3-hydroxyphenyl propionate (3-HPP), and 2- and 3-fluorobenzoate were also determined in the AWD5 genome. Hydroxyphenyl acetate (HPA) was found to be catabolized through meta-cleavage pathway. The strain showed its ability to degrade pyrene and benzo-a-pyrene more preferentially than chrysene. This indicates that AWD5 has broad potential for degradation of aromatic compounds. In the present study, *K. pneumoniae* AWD5 is revealed to enhance plant growth and contains genes conferring for IAA biosynthesis, phosphate solubilization, and siderophore production that are also supported by genome analysis. These unique characteristics of AWD5 genome suggest role of environmental variations reflected within the genome of a soil isolate that take up biodegradation of hydrocarbon in soil, and plant growth promotion, as its major physiological activities, as compared to other isolates of *K. pneumoniae*, which were clinical in nature. Therefore, *Klebsiella pneumoniae* AWD5 has the potential to be used for rhizoremediation of polyaromatic hydrocarbon-contaminated soil.

## Electronic supplementary material

Below is the link to the electronic supplementary material.

Supplementary material 1 (DOCX 18 kb)
