# Supplementary material for: Genomic insights of aromatic hydrocarbon degrading Klebsiella pneumoniae AWD5 with plant growth promoting attributes: a paradigm of soil isolate with elements of biodegradation
Source: 3 Biotech. 2018 Feb 7;8(2):118. doi: 10.1007/s13205-018-1134-1 (PMC5803133; doi:10.1007/s13205-018-1134-1)
Supplement: Supplementary file 1 — Supplementary material 1 (DOCX 18 kb) [file 13205_2018_1134_MOESM1_ESM.docx]

**Supplementary Data**

**Table S1**

Comparative summary of hydrocarbon degradation and other annotated gene functions of *K. pneumoniae* AWD5 genome with that of *K. pneumoniae* ATCC BAA-2146 (clinical, reference strain) and *K. pneumoniae* KP-1 (environmental isolate) genomes.

|  | **Genes** | **Function** | **Position** | | |
| --- | --- | --- | --- | --- | --- |
|  |  |  | **AWD5** | **ATCC BAA 2146 *** | **KP-1 *** |
| Phenyl acetic acid metabolism | *paaZ* | 3-oxo-5,6dehydrosuberyl-CoA semialdehyde dehydrogenase | 2873390-2875435 | 58892-60937 (99) | 74615-76660 (99) |
|  | *paaA* | Ring-1,2-phenylacetyl-CoA epoxidase subunit PaaA | 2875722-2876651 | 61224-62153 (99) | 76949-77876 (99) |
|  | *paaB* | Ring-1,2-phenylacetyl-CoA epoxidase subunit PaaB | 2876663-2876950 | 62165-62452 (99) | 77888-78175 (99) |
|  | *paaC* | Ring-1,2-phenylacetyl-CoA epoxidase subunit PaaC | 2876958-2877713 | 62460-63215 (99) | 78183-78938 (99) |
|  | *paaD* | Ring-1,2-phenylacetyl-CoA epoxidase subunit PaaD | 2877725-2878222 | 63227-63724 (98) | 78950-79447 (97) |
|  | *paaE* | Ring-1,2-phenylacetyl-CoA epoxidase subunit PaaE | 2878230-2879300 | 63732-64802 (100) | 79455-80525 (100) |
|  | *paaF* | Enoyl-CoA hydratase | 2879297-2880064 | 64799-65566 (99) | 80522-81281 (99) |
|  | *paaG* | Phenylacetate degradation probable enoyl-CoA hydratase | 2880667-2880855 | 65569-66357 (99) | 81292-82080 (99) |
|  | *paaH* | 3-hydroxybutyryl-CoA dehydrogenase | 2880859-2882280 | 66361-67782 (98) | 82084-83505 (99) |
|  | *paaI* | Phenyacetic acid degradation protein PaaD | 2882270-2882692 | 67772-68194 (100) | 83495-83917 (100) |
|  | *paaJ* | 3-oxo-5,6-didehydrosuberyl-CoA | 2882692-2883897 | 68194-89399 (99) | 83917-85122 (100) |
|  | *paaK* | Phenylacetate-CoA ligase | 2883924-2885240 | 69426-70742 (99) | 85149-86465 (99) |
| Aromatic degrading genes | *benC-xylZ* | Benzoate/toluate-1,2-dioxygenase reductase subunit | 3636407-3637423 | 157998-159014 (99) | 67378-68394 (98) |
|  | *benB-xylX* | Benzoate/toluate 1,2 dioxygenase beta subunit | 3637433-3637918 | 159024-159509 (99) | 68404-68889 (99) |
|  | *catA* | Catechol 1,2 dioxygenase | 3639410-3640336 | 161001-161927 (99) | 70381-71307 (99) |
|  | *catC* | Muconolactone delta-isomerase | 3640403-3640693 | 161994-162284 (99) | 71374-71664 (100) |
|  | *catB* | Muconate cycloisomerase | 3640713-3641831 | 162304-163422 (99) | 71684-72802 (99) |
|  | *ydcO* | Benzoate transporter | 1778084-1779250 | 229882-231048 (99) | 132374-133540 (99) |
|  | *pcaH* | Protocatechuate 3,4-dioxygenase, beta subunit | 1791815-1792555 | 243613-244353 (99) | 146104-146844 (99) |
|  | *pcaG* | Protocatechuate 3,4-dioxygenase, alpha subunit | 1792552-1793172 | 244350..244970 (99) | 146841-147461 (99) |
| Hydroxy phenyl propionate degradation | *mhpT* | MFS transporter, AAHS family, 3-hydroxyphenylpropionic acid transporter | 4600455..4601651 | - | 139365..140561 (99) |
|  | *mhpB* | 2,3-dihydroxyphenyl propionate 1,2-dioxygenase | 4605384-4606328 | - | 144294-145238 (99) |
|  | *mhpC* | 2-hydroxy-6-ketonona-2,4-dienedioate hydrolase | 4604500-4605366 | - | 143410-144276 (99) |
|  | *mhpA* | 3-hydroxyphenylpropionate hydroxylase | 4606330-4607994 | - | 145240-146904 (98) |
|  | *mhpD* | 2-keto-4-pentenoate hydratase | 4603683-4604489 | - | 142593-143399 (99) |
|  | *mhpF* | Acetaldehyde dehydrogenase | 4602740-4603690 | - | 141650-142600 (99) |
|  | *mhpE* | 4-hydroxy 2-oxovalerate aldolase | 4601727-4602743 | - | 140637-141653 (99) |
| 3, 4-dihydroxyphenylacetate degradation | *hpaC* | 4-hydroxyphenylacetate 3-monooxygenase reductase component | 1956775-1957287 | 34679-35191 (99) | - |
|  | *hpaB* | 4-hydroxyphenylacetate 3-monooxygenase oxygenase component | 1957307-1958869 | 35211-36773 (100) | - |
|  | *hpaA* | 4-hydroxyphenylacetate catabolism regulatory protein HpaA | 1959095-1959985 | 36999-37889 (99) | - |
|  | *hpaX* | 4-hydroxyphenylacetate permease | 1959995-1961353 | 37899-39257 (99) | - |
|  | *hpcH* | 2,4-dihydroxy hept-2-enedioate aldolase | 1961375-1962172 | 39279-40076 (99) | - |
|  | *hpaH* | 2 oxohept-3-enedioate hydratase | 1962183-1962986 | 40087-40890 (99) | - |
|  | *hpaF* | 5-carboxymethyl-2-hydroxymuconate delta-isomerase | 1963095-1963475 | 40999-41379 (100) | - |
|  | *hpaD/hpcB* | 3,4-dihydroxyphenylacatate 2,3-dioxygenase | 1963485-1964342 | 41389-42246 (99) | - |
|  | *hpcC* | 5-carboxymethyl-2-hydroxymuconate semialdehyde | 1964344-1965810 | 42248-43714 (100) | - |
|  | *hpaG* | 5-carboxy-2-oxohept-3-enedioate decarboxylase HpaG2 subunit | 1965807-1966571 | 43711-44475 (99) | - |
|  | *hpaG* | 5-carboxy-2-oxohept-3-enedioate decarboxylase HpaG1 subunit | 1966568-1967200 | 44472-45104 (99) | - |
|  | *hpaR* | Homoprotocatechuate degradative operon regulator | 1967487-1967927 | 45391-45831 (100) | - |
| Drug metabolism-cytochrome p450 | *Gst* | Glutathione S transferase | 1815670-1816275 | 267468-268073 (99) | 5449-6054 (99) |
|  | *yfcF* | Glutathione S transferase | 4556952-4557590 | 115827-116465 (99) | 449967-450605 (100) |
|  | *yliJ* | Glutathione S transferase | 2369611-2370237 | 83665-84291 (100) | 135528-136154 (99) |
| Transport and catabolism | *fadD* | Long chain acyl-CoA synthetase | 3961844-3963562 | 631510-633195 (99) | 27629-29347 (100) |
|  | *sodC* | Superoxide dismutase, Cu-Zn family | 1827019-1827540 | 278817-279338 (100) | 16798-17319 (100) |
| Multi drug resistance protein | *acrB* | Multidrug efflux pump | 1144563-1147709 | 303586-306732 (100) | 229687-232833 (100) |
|  | *arnT* | 4-amino-4-deoxy-L-arabinose transferase | 158078-159733 | 125578-127233 (99) | 125751-127406 (99) |
|  | *ampG* | MFS-transporter, PAT family, beta lactamase induction signal transducer | 1095369-1096844 | 254392-255867 (99) | 180493-181968 (99) |
| Infection related genes | *ureC* | Epithelial cell signaling as in *Helicobacter pyroli* infection | - | 617416-619119 | 108547-110250 |
|  | *Fim* | Major type 1 subunit fimbrin (pillin) | 3230782-3231363 | 93528-94104 (99) | 67040-67621 (100) |
|  | *fimA* | Major type 1 subunit fimbrin (pillin) | - | 84108-84656 | 57620-58168 |
|  | *pagP* | Palmitoyl transferase | - | 104793-105311 | 531133-531651 |
|  | *norV* | Anaerobic nitric oxide flavoruvredoxin | - | 143748-145196 | 117260-118708 |
|  | *norW* | Nitric oxide reductase FIRd-NAD (+) reductase | 3282447-3283580 | 145193-146326 (99) | 118705-119838 (99) |

* Percentage similarity of annotated sequence with respective annotated gene sequence of *K. pneumoniae* AWD5 is given in parenthesis next to gene position of *K. pneumoniae* ATCC BAA 2146, and *K. pneumoniae* KP-1, as determined by BLAST analysis.
